# BnaC04.bZIP16 can be phosphorylated and inhibited by BnaA06.SnRK2 and negatively regulates the accumulation of fatty acids in *Brassica napus*

**DOI:** 10.1111/tpj.70506

**Published:** 2025-10-03

**Authors:** Wei Hui, Shuangshuang Li, Jinhao Ding, Qianru Li, Yuhong Chen, Yanhui Wang, Xupeng Guo, Chengming Fan, Zanmin Hu

**Affiliations:** ^1^ Key Laboratory of Seed Innovation, Institute of Genetics and Developmental Biology, Innovation Academy for Seed Design Chinese Academy of Sciences Beijing 100101 China; ^2^ College of Advanced Agricultural Sciences University of Chinese Academy of Sciences Beijing 100049 China; ^3^ Leshan Academy of Agricultural Sciences Leshan Sichuan China

**Keywords:** BnaC04.bZIP16, fatty acid biosynthesis, BnaA06.SnRK2, phosphorylation, *Brassica napus*

## Abstract

*Brassica napus* is an important oil crop with a significant amount of fatty acid in its seeds. However, the molecular mechanisms of fatty acid synthesis and accumulation are still poorly understood. In this study, we identified a transcription factor, *BnaC04.bZIP16*, which belongs to the G subfamily of the bZIP family, that negatively regulates the accumulation of fatty acid in *B. napus*. BnaC04.bZIP16 directly binds to the promoters of *BnaA08.OLEOSIN1* and *BnaC03.PDH‐E1 BETA* to regulate seed fatty acid content. Furthermore, BnaC04.bZIP16 can be phosphorylated by the BnaA06.SnRK2. BnaA06.SnRK2‐dependent phosphorylation can weaken BnaC04.bZIP16 protein stability as well as the DNA‐binding ability. Interestingly, overexpression of *BnaA06.SnRK2* can significantly promote the accumulation of fatty acid. Our study identified a new function of *BnaC04.bZIP16* in regulating the accumulation of fatty acids in *B. napus* and added it to the G subfamily of the bZIP family. Furthermore, our study proposed a regulatory pathway, ‘BnaA06.SnRK2‐BnaC04.bZIP16‐*BnaA08.OLEOSIN1*/*BnaC03.PDH‐E1 BETA*’, which provides novel insight into fatty acid biosynthesis.

## Significance Statement

Although key enzymes in fatty acid metabolism have been extensively studied, the upstream regulatory pathways remain largely unclear in *Brassica napus*. This study uncovers a transcription factor and kinase module that fine‐tunes fatty acid accumulation during seed development, providing new insights into the transcriptional regulation of fatty acid biosynthesis in oilseed crops.

## INTRODUCTION

*Brassica napus* is the third largest source of vegetable oil in the world (Tang et al., 2021), accounting for approximately 12.7% of global edible oil production. Triacylglycerol (TAG) is the main component of vegetable oils (Wolff, 1966), which is a glycerol ester produced by the esterification of fatty acid (FA) attached to three molecules on the glycerol skeleton. The accumulation of TAG begins with de novo synthesis of FA in the plastid, followed by TAG assembly on the endoplasmic reticulum (Kennedy & Weiss, 1956; Li‐Beisson et al., 2013; Xu & Shanklin, 2016).

The synthesis and accumulation of fatty acids are regulated by a multigene regulatory network in which transcription factors (TFs) play an important biological function in the regulation of fatty acid biosynthesis. To date, TFs from several families—such as AP2/EREBP, B3, NF‐Y, MYB, and WRKY—have been extensively studied. For example, WRI1, a member of the AP2/EREBP family, has been identified as a central regulator of fatty acid synthesis, and its overexpression has been shown to significantly enhance oil content (Baud & Lepiniec, 2009; Kuczynski et al., 2022; Maeo et al., 2009). The LAFL network, including LEAFY COTYLEDON1 (LEC1), ABSCISIC ACID INSENSITIVE3 (ABI3), FUSCA3 (FUS3), and LEAFY COTYLEDON2 (LEC2), also plays a pivotal role in regulating the seed fatty acid content. *FUS3*, *LEC2*, and *ABI3* encode B3‐domain‐containing TFs, and *LEC1* encodes the NF‐YB subunit of the NF‐Y family (Mu et al., 2013). Overexpression of *LEC1* upregulates the expression of *WRI1, FUS3, and ABI3*, thereby activating fatty acid biosynthesis (Santos Mendoza et al., 2005; Vicient et al., 2000). Furthermore, LEC2 directly targets WRI1, and fatty acid content is reduced by 30% in *lec2* mutant seeds (Angeles‐Nunez & Tiessen, 2011). TFs from the MYB family—particularly R2R3‐MYB (Stracke et al., 2001)—also play diverse roles in the accumulation of oil. While *MYB123* (*TT2*) (Wang et al., 2014), *MYB118* (Barthole et al., 2014), *MYB89* (Li et al., 2017), and *MYB76* (Duan et al., 2017) negatively regulate fatty acid synthesis, *MYB92* (To et al., 2020), *MYB96* (Lee et al., 2018), *MYB30* (Raffaele et al., 2008), and *MYB108* (Xu et al., 2022) act as positive regulators. Similarly, in the WRKY family, *WRKY6* significantly increased the accumulation of fatty acids in *Arabidopsis thaliana* seeds (Song et al., 2020), and *WRKY43* modulates unsaturated fatty acid content under stress conditions (Geilen et al., 2017).

In contrast to these well‐characterized families, research on the bZIP transcription factor family in fatty acid biosynthesis remains limited. The bZIP family, particularly the G subfamily, is best known for its role in seed germination (Zinsmeister et al., 2016), organ growth (Wang et al., 2020), biomass accumulation (Zhang et al., 2015), and flowering time control in plants (Jakoby et al., 2002; Mallappa et al., 2008; Singh et al., 2012; Terzaghi et al., 1997; Wigge et al., 2005). Functional studies of bZIP TFs in lipid metabolism are rare and mostly limited to A subfamily members, such as ABI5 and bZIP67, which regulate oil content and FA composition (Mendes et al., 2013; Yeap et al., 2017). Thus, the role of G subfamily bZIPs in seed FA biosynthesis remains largely unexplored.

In this study, we identified six bZIP16 homologs in the *B. napus* genome based on sequence similarity to *Arabidopsis* bZIP16. Among them, BnaC04.bZIP16 was found to be highly expressed in developing siliques and seeds, suggesting a potential role in seed lipid metabolism. We therefore selected BnaC04.bZIP16 for detailed functional and mechanistic analysis. Using overexpression and CRISPR/Cas9 knockout approaches, we demonstrated that BnaC04.bZIP16 negatively regulates seed FA accumulation. Furthermore, we discovered that BnaA06.SnRK2 phosphorylates BnaC04.bZIP16, modulating its function in regulating FA biosynthetic genes. Our findings uncover a previously uncharacterized bZIP‐mediated regulatory pathway and establish a mechanistic link between SnRK2 signaling and transcriptional control of FA biosynthesis in *B. napus* seeds.

## RESULTS

### The overexpression of *BnaC04.bZIP16* decreased the fatty acid content in *A. thaliana*

**Figure 1:**
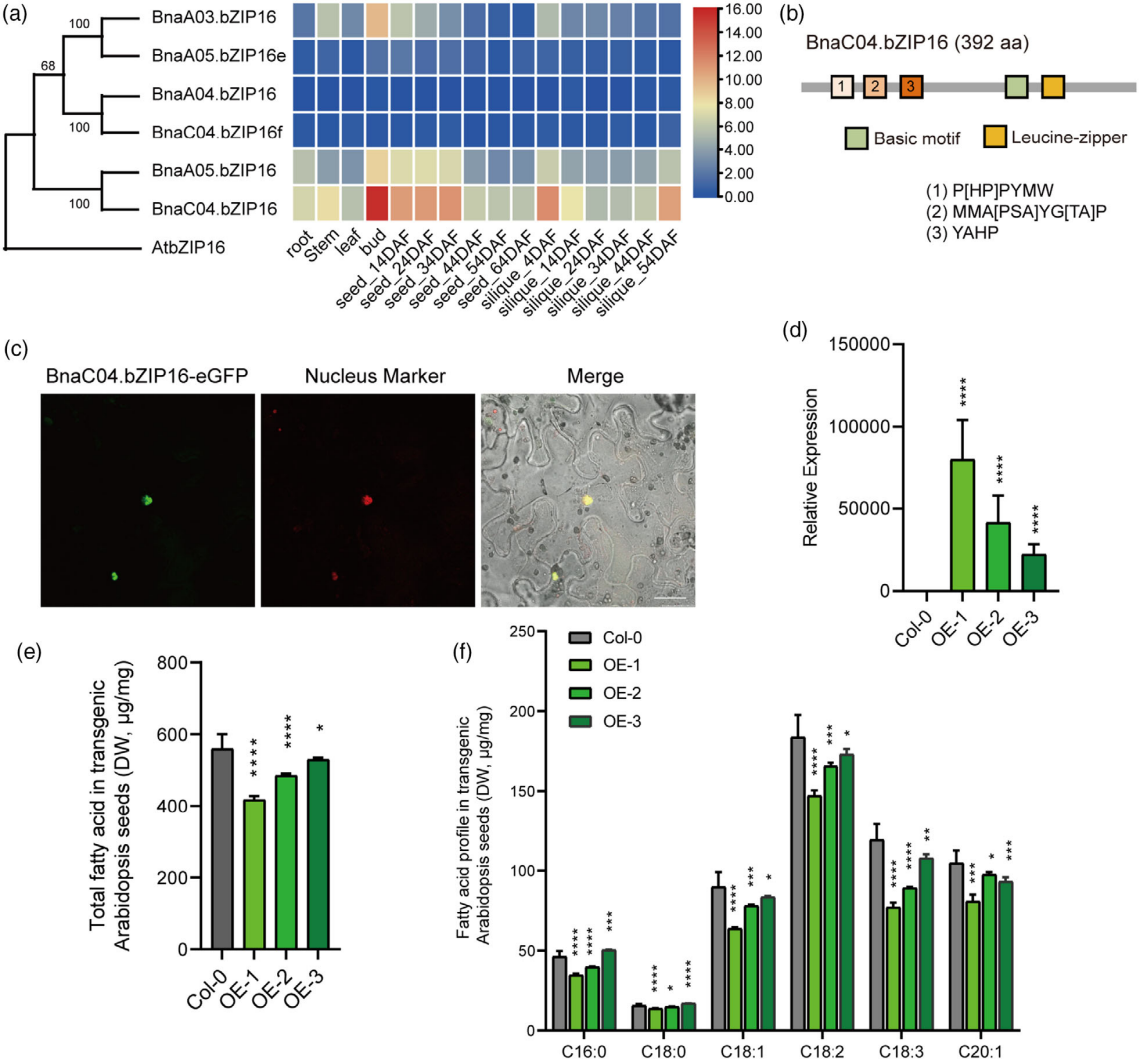
Molecular characteristics of BnaC04.bZIP16 and effects of overexpression of *BnaC04.bZIP16* on seed fatty acid content in transgenic *A. thaliana*. (a) Tissue‐specific expression pattern of *BnabZIP16s* in *B. napus* tissues. The data were from BnTIR (http://yanglab.hzau.edu.cn/BnTIR) and TAIR (https://www.Arabidopsis.org/). (b) Schematic representation of the BnaC04.bZIP16 protein structure. 1, 2, and 3 represent judgment basis for determining the G subfamily of bZIP family in *Arabidopsis* and *B. napus*. (c) Subcellular localization of BnaC04.bZIP16 in tobacco cells. Green is the BnaC04.bZIP16‐eGFP signal and red is the nuclear marker signal. Scale bar, 25 μm. (d) Expression level of *BnaC04.bZIP16‐eGFP* in *Arabidopsis*. eGFP primers were used to perform qPCR, *AtActin2* (AT3G18780) was used as a control for normalization. (e, f) Total fatty acid content and fatty acid profile in *BnaC04.bZIP16*‐transgenic *Arabidopsis* seeds, respectively. Error bars indicate SD (*n* = 4). Asterisks indicate significant differences (****P < 0.001; ***P < 0.005; **P < 0.01; *P < 0.05. Student's *t* test).

To elucidate the function of bZIP16 in *B. napus*, the amino acid sequences of *Arabidopsis* bZIP16 were used for BLAST analysis in the BnTIR database (https://yanglab.hzau.edu.cn/BnTIR). The results show that there are six homologous copies of *bZIP16* (BnaC04G0109700ZS, designated as *BnaC04.bZIP16*; BnaA05G0091400ZS, designated as *BnaA05.bZIP16*; BnaA03G0169600ZS, designated as *B* *naA03.bZIP16*; BnaA04G0226300ZS, designated as *BnaA04.bZIP16*; BnaC03G0196500ZS, designated as *BnaA05.bZIP16e*; and BnaC04G0540300ZS, designated as *BnaC04.bZIP16f*). Subsequently, we retrieved the expression levels of *BnabZIP16* in different tissues from the BnTIR database (Liu et al., 2021). The results showed that the expression levels of *BnaA03.bZIP16*, *BnaA04.bZIP16*, *BnaA05.bZIP16e*, and *BnaC04.bZIP16f* are very low in different tissues, while *BnaC04.bZIP16* and *BnaA05.bZIP16* have higher expression in developing siliques and seeds, especially *BnaC04.bZIP16* (Figure 1a, Figure S1). BnaC04.bZIP16 protein has 392 amino acid residues, including a basic motif and a leucine zipper motif, and possesses three conserved sequences preceding the basic motif, aligning with the characteristics of the G subfamily of the bZIP family (Figure 1b, Figure S1).

BnaC04.bZIP16 fused with GFP at its C‐terminus was located in the cell nucleus, determined by its transient expression in the epidermal cells of tobacco and stable expression in *Arabidopsis* (Figure 1c, Figure S1). The results were consistent with the classical characteristics of transcription factors.

*BnaC04.bZIP16* having a notably higher expression level than the other five homologous genes in developing siliques and seeds was overexpressed driven by the 35S promoter in *Arabidopsis* plants (Figure 1d). Three homozygous transgenic lines with various *BnaC04.bZIP16* expression levels were evaluated for fatty acid (FA) content of seeds. Compared with wild‐type plants (Col‐0), all the *OE‐BnaC04.bZIP16 Arabidopsis* seeds showed a lower total FA content of 5%–25% by dry weight (Figure 1e). The contents of fatty acid compositions were also significantly reduced compared to those in Col‐0, especially unsaturated fatty acids (Figure 1f). These results indicate that *BnaC04.bZIP16* negatively regulates fatty acid accumulation, which is worthy of further study.

### The knockout of *BnaC04.bZIP16* increased the fatty acid content in *B. napus*

**Figure 2:**
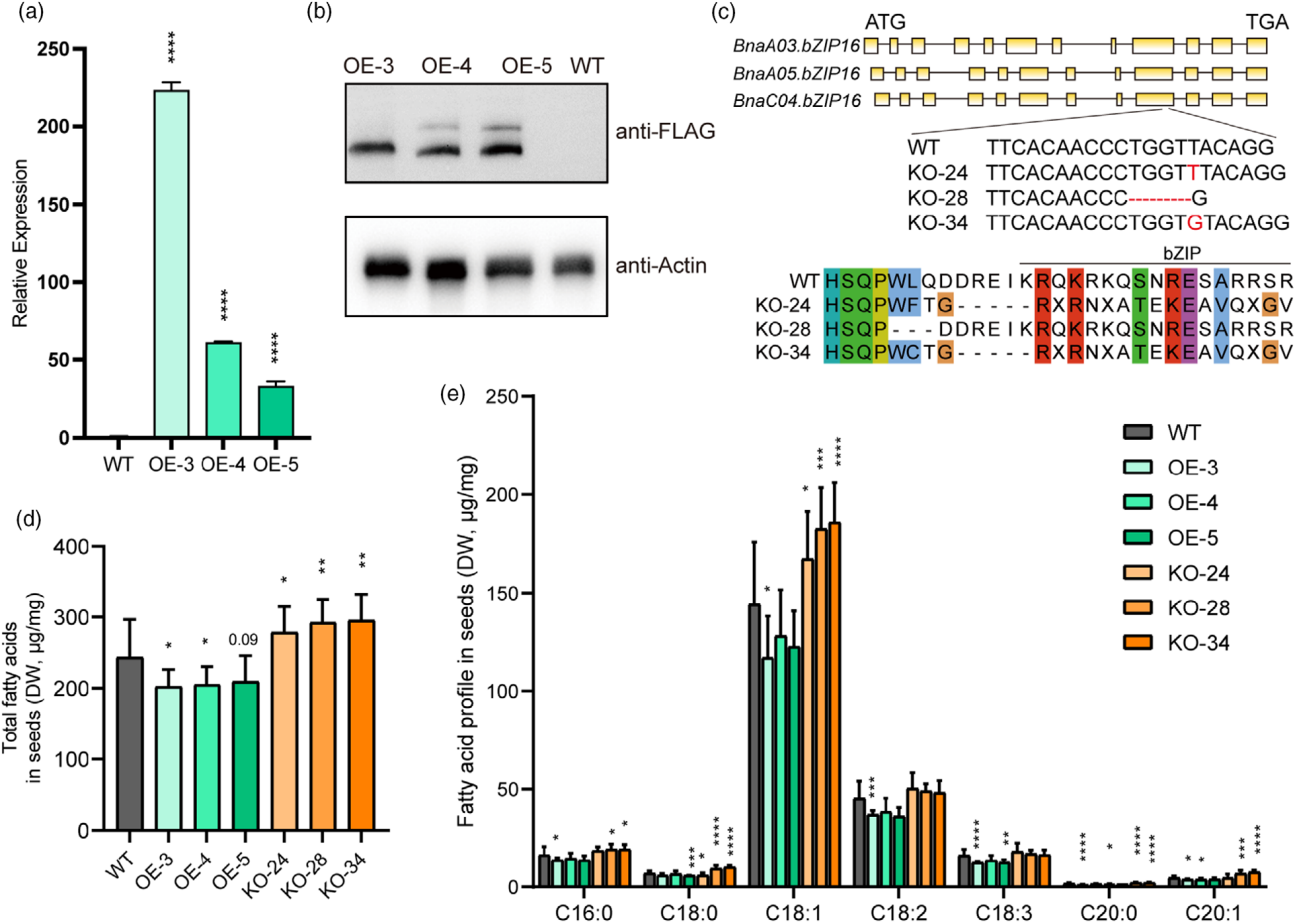
*BnaC04.bZIP16* negatively regulates the fatty acid content in *Brassica napus*, which is shown by its overexpression and knockout plants. (a) Relative expression level of *BnaC04.bZIP16* in OE lines by qRT‐PCR. Total RNA was extracted from 15 DAF seeds. *BnaC02.Actin7* (BnaC02g00690D) was used as a control for normalization. Error bars indicate SD (*n* = 3). (b) Expression of the BnaC04.bZIP16 in OE lines by Western blot using Flag antibody. Protein was extracted from 15 DAF seeds. (c) Acquisition of mutant lines by CRISPR–Cas9‐based genome editing. CRISPR targets the ninth exon of *BnaC04.bZIP16*, *BnaA05.bZIP16* and *BnaA03.bZIP16*. KO‐24, KO‐28 and KO‐34 are homozygous three mutants. (d, e) Total fatty acid content and fatty acid profile in *BnaC04.bZIP16*‐transgenic *B. napus* seeds. Error bars indicate SD (*n* = 5). OE represents overexpression and KO represents knockout. Asterisks indicate significant differences compared to the control wild‐type plants (****P < 0.001; ***P < 0.005; **P < 0.01; *P < 0.05. Student's *t* test).

To further demonstrate the function of *BnaC04.bZIP16* in *B. napus*, we generated *35S::BnaC04.bZIP16‐FLAG* stable transgenic overexpressed *B. napus* (*OE‐BnaC04.bZIP16*) under the background of *B. napus* cv. Westar. Three transgenic lines with increased expression of *BnaC04.bZIP16* in the seeds at 15 days after flowering (DAF) are successfully obtained (Figure 2a). Western blot using Flag antibody confirms that positive OE plants are acquired (Figure 2b). CRISPR‐Cas9 was utilized to create mutants of BnabZIP16s. As a result, three homozygous knockout mutants KO‐24, KO‐28, and KO‐34 with mutations in *BnaC04.bZIP16, BnaA05.bZIP16*, and *BnaA03.bZIP16* are identified by sequencing (Figure 2c, Figure S2a). The transgenic lines of *BnaC04.bZIP16* do not exhibit adverse agronomic traits (Figure S2b). Pollen staining showed that *OE‐BnaC04.bZIP16* did not affect pollen viability (Figure S2c).

The total fatty acid content of OE, KO, and WT seeds was measured. Compared with wild type, the fatty acid content of seeds of OE lines was 15%–17.9% lower, and the fatty acid content of seeds of KO lines was 12.9%–19.7% greater (Figure 2d). Furthermore, we compared the main fatty acid compositions; the content of some compositions significantly changed, especially that of oleic acid (C18:1), which was significantly higher in KO lines compared with that of WT (Figure 2e). We further measured the content of soluble sugars and total proteins, both of which were slightly reduced or remained unchanged in the KO line compared to the WT (Figure S2d,e). In addition, there were no significant differences in seed size (length, width, and area) among the OE, KO, and WT seeds (Figure S2f). The yield per plant of the KO lines was slightly increased or remained unchanged compared to the WT (Figure S2g). These results indicated that *BnaC04.bZIP16* negatively regulated fatty acid content in *B. napus* seeds.

### *BnaC04.bZIP16* regulates the expression of fatty acid biosynthesis‐related genes

**Figure 3:**
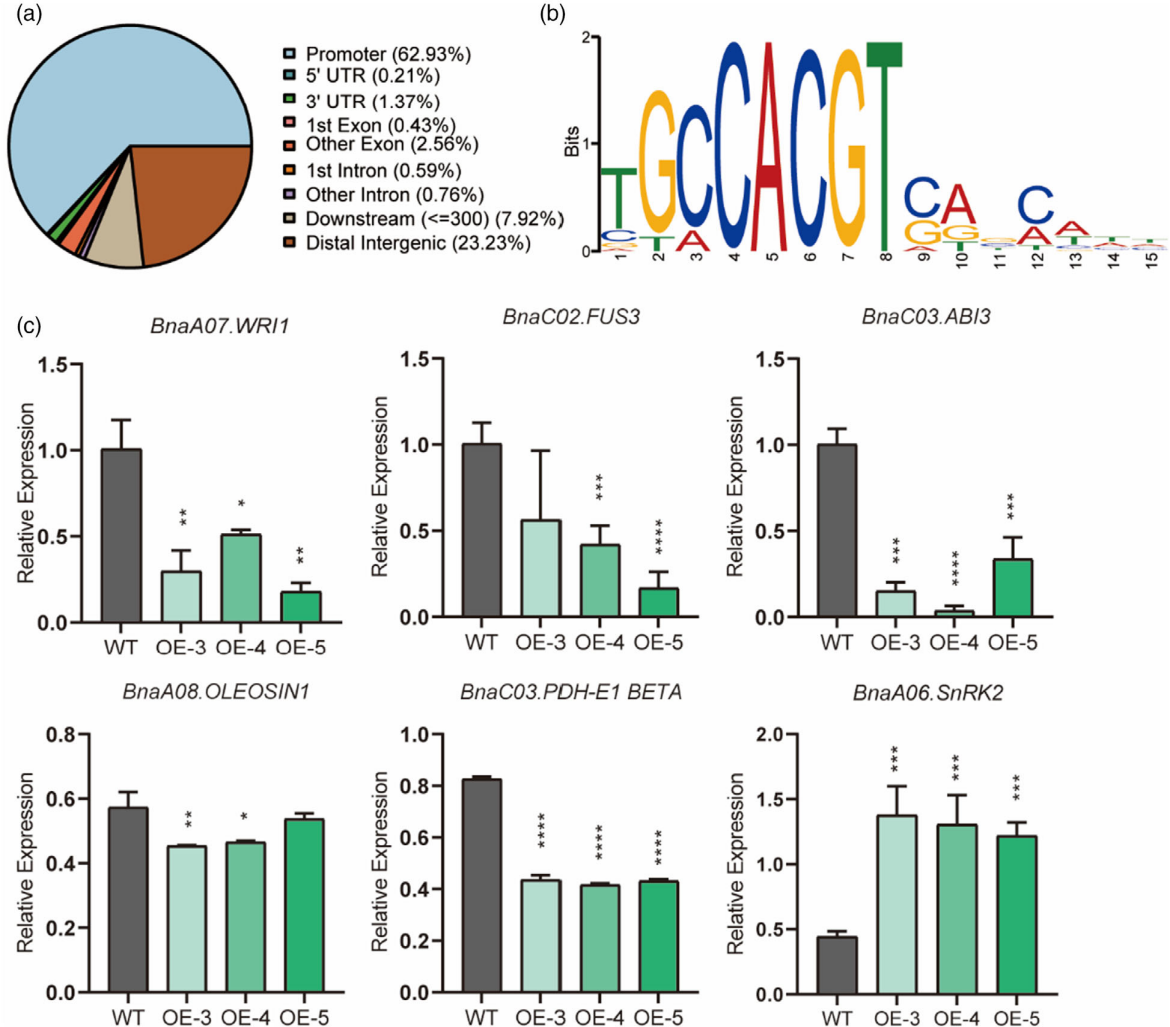
*BnaC04.bZIP16* affects the expression of fatty acid metabolic pathway genes. (a) Sequence types enriched by CUT&Tag in *OE‐BnaC04.bZIP16*; the blue section represents the promoter. (b) The core sequence of CUT&Tag enriched sequences is obtained after MEME‐ChIP analysis (https://meme‐suite.org/meme/tools/meme‐chip). (c) Relative expression of *BnaA07.WRI1*, *BnaC02.FUS3*, *BnaC03.ABI3*, *BnaA08.OLEOSIN1*, *BnaC03.PDH‐E1 BETA* and *BnaA06.SnRK2* in WT and OE lines in *B. napus*. Total RNA was extracted from 15 DAF seeds. Error bars indicate SD (*n* = 3). Asterisks indicate significant differences compared to the control wild type (****P < 0.001; ***P < 0.005; **P < 0.01; *P < 0.05. Student's *t* test).

To identify the direct regulatory target genes regulated by BnaC04.bZIP16 protein during fatty acid accumulation, CUT&Tag analysis was performed using 15 DAF siliques of *OE‐BnaC04.bZIP16* lines. The results showed that 62.93% of high‐confidence binding peaks (3072) were located in promoter regions of annotated genes in the *B. napus* genome (Figure 3a). Motif enrichment analysis using MEME‐ChIP (https://meme‐suite.org/meme/) identified the canonical G‐box sequence (CACGT), a known bZIP binding motif, as the most enriched site (Figure 3b). GO enrichment analysis (Zhou et al., 2019) of the bound genes indicated significant enrichment in biological processes related to seed development, carbohydrate metabolism, and fatty acid biosynthesis (Figure S4). Based on the result, we selected 10 candidate genes involved in FA metabolism whose promoters were bound by *BnaC04.bZIP16* in the CUT&Tag dataset such as *BnaA08.OLEOSIN1* (BnaA08g14540D), *BnaA08.SPH2* (BnaA08g09710D), *BnaC02.PKT1_KAT5* (BnaC02g38800D), *BnaC09.CLS* (BnaC09g22540D), *BnaC02.DGK1* (BnaC02g01400D), *BnaC05.DGD1* (BnaC05g41530D), *BnaA06.FATB* (BnaA06g04900D), *BnaC07.ATPXG2* (BnaC07g27360D), *BnaC03.PDH‐E1 BETA* (BnaC03g59130D), and *BnaA06.DSI‐1VOC* (BnaA06g04170D). The 1.5 kb sequences upstream of the ATG start codon of these genes were cloned and subjected to dual‐luciferase reporter assays (Figure S5a,b). These results suggest that BnaC04.bZIP16 functions as a negative regulator of fatty acid accumulation, which was consistent with Figure 3c.

To further support this, we performed qRT‐PCR using 15 DAF seeds from WT and OE‐*BnaC04.bZIP16* lines and observed significantly reduced expression of several fatty acid‐related genes, including *BnaA07.WRI1* (BnaA07g16350D), *BnaC02.FUS3* (BnaC02g36350D), *BnaC03.ABI3* (BnaC03g44820D), *BnaA08.OLEOSIN1* (BnaA08g14540D), and *BnaC03.PDH‐E1 BETA* (BnaC03g59130D) (Figure 3c). Together, these findings indicate that BnaC04.bZIP16 regulates fatty acid accumulation through both direct and indirect mechanisms.

**Figure 4:**
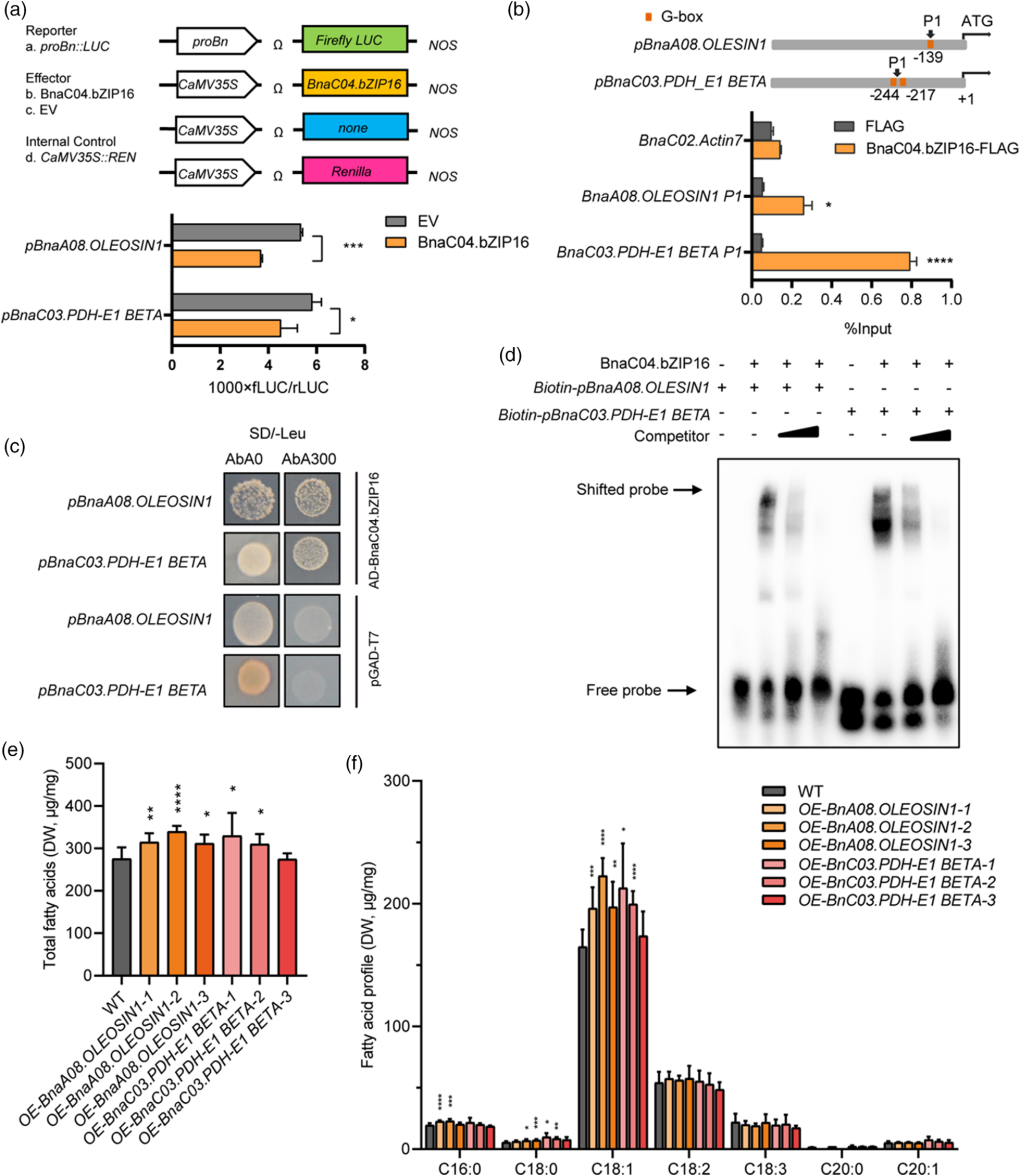
BnaC04.bZIP16 directly regulates the expression levels of *BnaA08.OLEOSIN1* and *BnaC03.PDH‐E1 BETA*. (a) Dual‐LUC assay in *Nicotiana* leaves. Effectors: *35S::NOS* and *35S::BnaC04.bZIP16*. (b) ChIP assay showing the binding of BnaC04.bZIP16 to the promoters of *BnaA08.OLEOSIN1* and *BnaC03.PDH‐E1 BETA in vivo*. ChIP was performed with an anti‐FLAG antibody. The immunoprecipitated DNA was quantified by qPCR using primers specific to regions within the *BnaA08.OLEOSIN1* and *BnaC03.PDH‐E1 BETA* loci P1, which contains canonical G‐box motifs. Relative enrichment is represented as %Input, and the data are the means ± SD (*n* = 3). (c) Y1H assay on the binding of BnaC04.bZIP16 protein to the promoters of *BnaA08.OLEOSIN1* and *BnaC03.PDH‐E1 BETA*. SD/−Leu, synthetic defined medium lacking leucine; AbA, aureobasidin A, working concentration 300 ng/mL. pGADT7 was used as a negative control. Effectors: *35S::NOS* and *35S::BnaC04.bZIP16‐NOS*; Reporter: *proBnaA08.OLEOSIN1/proBnaC03.PDH‐E1 BETA::LUC*; Internal control: *35S::REN*. Error bars indicate SD (*n* = 3). Asterisks indicate significant differences compared to the control (****P < 0.001; ***P < 0.005; **P < 0.01; *P < 0.05. Student's *t* test). (d) EMSA of the specific binding of His‐BnaC04.bZIP16 to the promoters P1 region of *BnaA08.OLEOSIN1* and *BnaC03.PDH‐E1 BETA*. The biotin‐labeled probes were designed to target the G‐box, and the amounts of competing probes were 10 and 50 times higher than those of the labeled probes. (e, f) Total fatty acid content and fatty acid profile in *BnaA08.OLEOSIN1* and *BnaC03.PDH‐E1 BETA* transgenic *B. napus* seeds. Error bars indicate SD (*n* = 5). Asterisks indicate significant differences compared to the control wild‐type plants (****P < 0.001; ***P < 0.005; **P < 0.01; *P < 0.05. Student's *t* test).

Among the downregulated targets, *BnaA08.OLEOSIN1* (Oil Body Protein) and *BnaC03.PDH‐E1 BETA* (Pyruvate Dehydrogenase) were selected for further validation due to their well‐established roles in oil body formation and acetyl‐CoA generation, respectively. Additionally, both genes showed strong and consistent regulation in expression and luciferase assays (Figure 4a, Figure S5), making them representative downstream effectors for functional characterization of *BnaC04.bZIP16*.

### *BnaC04.bZIP16* directly regulates its downstream genes for fatty acid biosynthesis

To verify whether BnaC04.bZIP16 can directly regulate the expression of *BnaA08.OLEOSIN1* and *BnaC03.PDH‐E1 BETA*, we analyzed the 1.5 kb sequences upstream of the ATG start codon of *BnaA08.OLEOSIN1* and *BnaC03.PDH‐E1 BETA* genes. Motif analysis showed that there was at least one G‐box motif in their promoters (Figure 4b). Furthermore, Chromatin immunoprecipitation (ChIP) assay was performed with anti‐FLAG antibody using 15 DAF siliques of *OE‐BnaC04.bZIP16‐FLAG* transgenic *B. napus*, followed by q‐PCR. ChIP‐qPCR demonstrated that BnaC04.bZIP16‐FLAG was bound to the P1 site (CACGTG) of the *BnaA08.OLEOSIN1* and *BnaC03.PDH‐E1 BETA* promoters. Then, yeast one‐hybrid assay was performed using the 1.5 kb promoters of *BnaA08.OLEOSIN1/BnaC03.PDH‐E1 BETA* and *pGAD‐BnaC04.bZIP16*; yeast could grow on Leu‐deficient medium supplemented with Aureobasidin A (AbA) (Figure 4c). Furthermore, Electrophoretic mobility shift assay (EMSA) suggested that His‐BnaC04.bZIP16 directly binds to the P1 site in the *BnaA08.OLEOSIN1* and *BnaC03.PDH‐E1 BETA* promoters. Increasing amounts of unlabelled competing probes markedly reduced the binding of BnaC04.bZIP16 to biotin‐labeled probes (Figure 4d). These results showed that BnaC04.bZIP16 indeed directly binds to the promoters of *BnaA08.OLEOSIN1* and *BnaC03.PDH‐E1 BETA*.

Since BnaC04.bZIP16 directly regulates the expression of *BnaA08.OLEOSIN1* and *BnaC03.PDH‐E1 BETA*, transgenic plants with stable overexpression of *BnaA08.OLEOSIN1* and *BnaC03.PDH‐E1 BETA* were generated under the background of *B. napus* cv. Westar. The total fatty acid content of seeds in *OE‐BnaA08.OLEOSIN1* and *OE‐BnaC03.PDH‐E1 BETA* increased by 13%–23% and 13%–20%, respectively, compared with wild‐type (Figure 4e). The content of major fatty acid compositions of *OE‐BnaA08.OLEOSIN1* and *OE*‐*BnaC03.PDH‐E1 BETA*, especially oleic acid, was significantly increased compared with that of wild‐type seeds (C18:1) (Figure 4f).

### BnaA06.SnRK2 interacts with and phosphorylates BnaC04.bZIP16

**Figure 5:**
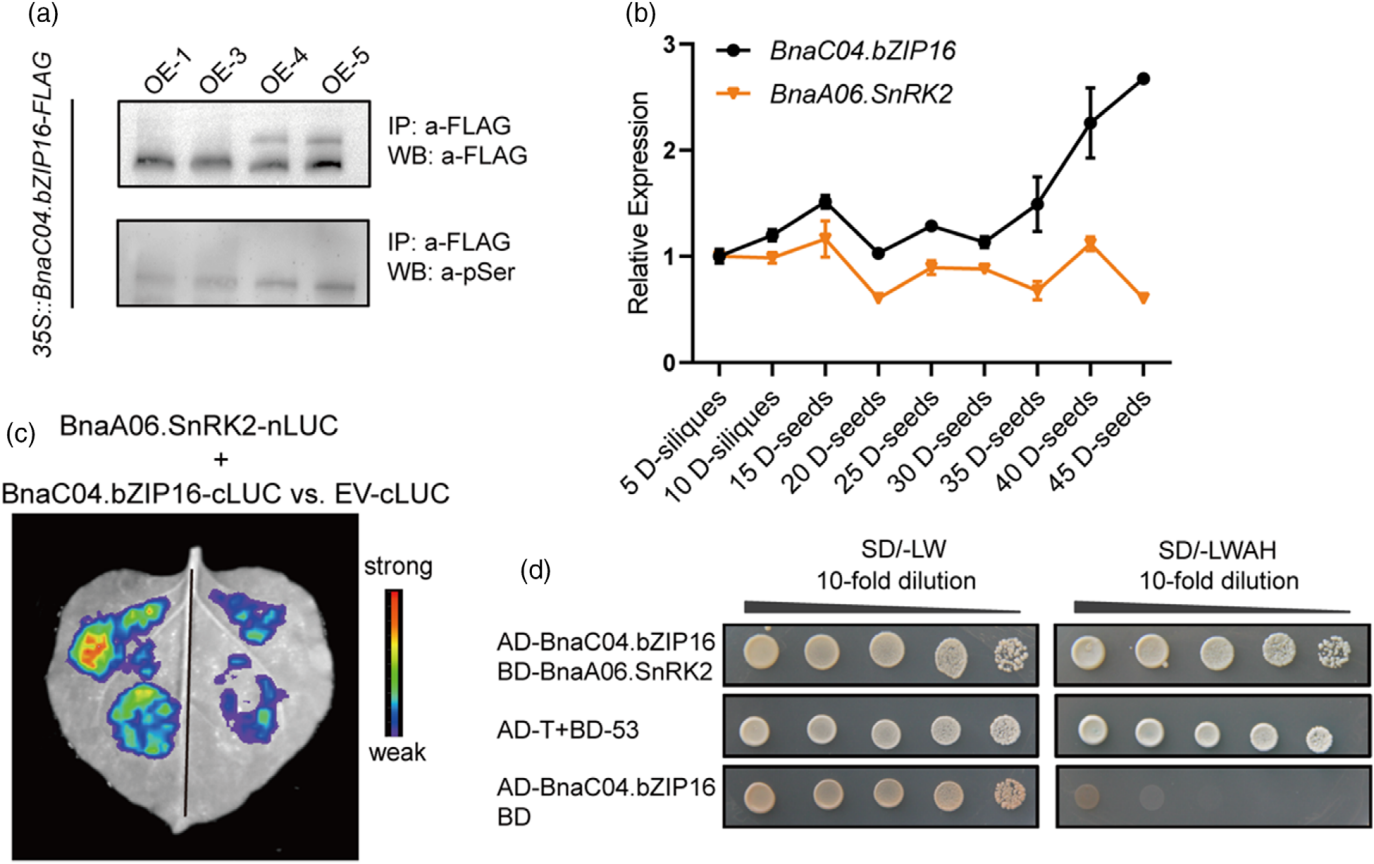
BnaA06.SnRK2 interacts with BnaC04.bZIP16. (a) BnaC04.bZIP16 can be phosphorylated at a serine site *in vivo*. Fifteen DAF seeds harboring the *35S::BnaC04.bZIP16‐FLAG* construct were immunoprecipitated with anti‐FLAG antibody, and the BnaC04.bZIP16‐FLAG proteins were immunoblotted against anti‐FLAG (top panel) and anti‐phosphoserine (a‐pSer) antibodies (bottom panel). (b) Expression patterns of *BnaA06.SnRK2* in different stages of *B. napus* seed development. (c) Split luciferase complementation assay. Agrobacterium cells containing plasmids *cLUC‐BnaC04.bZIP16* and *nLUC‐BnaA06.SnRK2* were used to transform tobacco and analyze the interaction between BnaC04.bZIP16 and BnaA06.SnRK2. (d) Yeast two‐hybrid assay. Yeast cells cotransformed with *AD‐BnaC04.bZIP16* and *BD‐BnaA06.SnRK2* constructs were grown on selective media. BD, pGBKT7; AD, pGADT7; SD/‐LW, synthetic defined medium lacking Leu and Trp; SD/‐LWAH, synthetic defined medium lacking Leu, Trp, Ade and His. *pGADT7‐T + pGBKT7‐53* was used as a positive control, and *pGADT7‐ BnaC04.bZIP16 + pGBKT7* was used as a negative control. Each clone was diluted 10^0^–10^4^ times.

As members of the bZIP family can usually be phosphorylated and affect their own functions (Meng et al., 2023). Immunoprecipitation (IP) assay was performed using 15 DAF seeds of *OE‐BnaC04.bZIP16‐FLAG* with anti‐FLAG antibody, followed by immunoblotting with an anti‐phosphoserine antibody (anti‐pSer). The phosphorylated bands were detected at the correct molecular weight for BnaC04.bZIP16‐FLAG protein (47.1 kDa), and the results indicated that serine of BnaC04.bZIP16 protein could be phosphorylated in *B. napus* (Figure 5a). Then, liquid chromatography–tandem mass spectrometry assay (LC–MS/MS) was conducted on the protein obtained after immunoprecipitation. A strong phosphorylation signal was detected in Ser151 of BnaC04.bZIP16 protein and this site is highly conserved in multiple species such as *A. thaliana*, *B. napus*, *Triticum aestivum*, *Oryza sativa*, *Brassica rapa*, *Brassica oleracea*, and *Glycine max* et al. (Figure S6a). In addition, 3811 proteins were detected that may interact with BnaC04.bZIP16 in LC–MS/MS; BnaA06g22800D (*BnaA06.SnRK2*) was selected as a potential interacting protein based on the *P* value and gene function as serine/threonine kinases, which is a SNF1‐related protein kinase and has higher identity with AtSnRK2.5 (AT5G63650) (Figure S6b).

The expression pattern of *BnaA06.SnRK2* was highly coincident with *BnaC04.bZIP16* before 45 DAF (Figure 5b). Besides, BnaA06.SnRK2 is mainly located in the nucleus and cytoplasm, which partially overlaps with that of BnaC04.bZIP16 (Figure S6c). These suggest a potential for molecular interactions between the two proteins. To further confirm the interaction between BnaA06.SnRK2 kinase and BnaC04.bZIP16 protein, a split luciferase complementation assay in *N. benthamiana* was performed; the reconstitution of yellow fluorescence protein was observed in the nucleus when co‐infiltrated with *BnaA06.SnRK2‐cLUC* and *BnaC04.bZIP16‐nLUC* constructs (Figure 5c). Bimolecular fluorescence complementation assay (BiFC) also showed the same result (Figure S6d). Next, a yeast two‐hybrid assay was performed using *pGADT7‐BnaC04.bZIP16* and *pGBKT7‐BnaA06.SnRK2* constructs. Y2HGold cells could grow on the medium lacking Leu, Trp, Ade, and His (Figure 5d). These results indicated that BnaA06.SnRK2 can interact with BnaC04.bZIP16.

### BnaA06.SnRK2‐mediated phosphorylation of BnaC04.bZIP16 attenuates its binding to the target gene promoter

**Figure 6:**
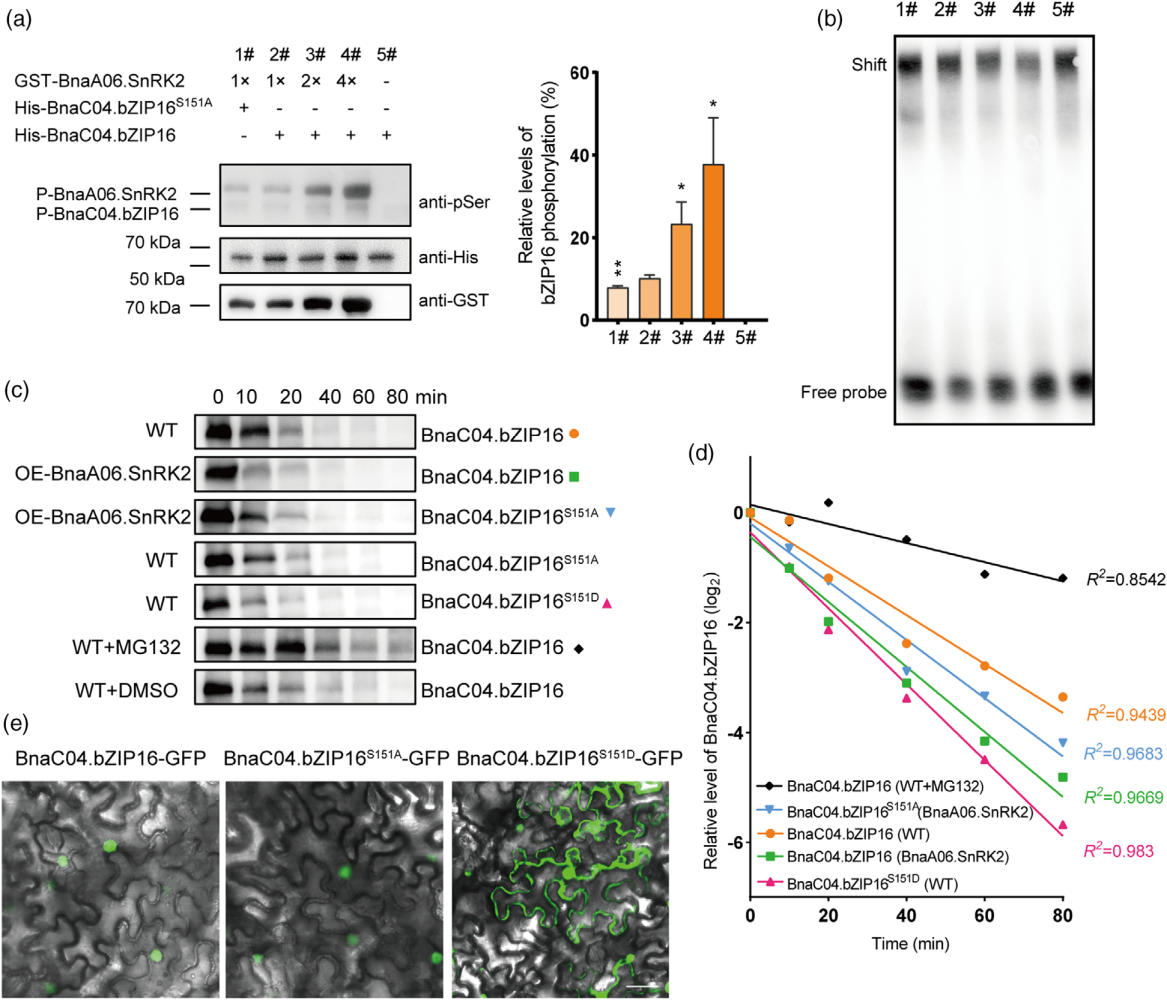
BnaA06.SnRK2‐mediated phosphorylation of BnaC04.bZIP16 attenuates its binding to DNA. (a) The kinase assay *in vitro*. Recombinant proteins were detected using anti‐pSer (top), anti‐His (middle), and anti‐GST (bottom) antibodies. His‐BnaC04.bZIP16 protein alone was used as a negative control. S151A the serine at position 151 is mutated to alanine. His‐BnaC04.bZIP16 (middle) was used as a calibration internal reference. Asterisks indicate significant differences compared to 2# (**P < 0.01; *P < 0.05. Student's *t* test). (b) EMSA showing the binding strength of phosphorylated His‐BnaC04.bZIP16 to the *BnaA08.OLEOSIN1* promoter. The protein obtained from the *in vitro* kinase assay shown in (a) was used in the experiment with a biotin‐labeled probe designed for the P1 region of the *BnaA08.OLEOSIN1* promoter. (c) *In vitro* cell‐free degradation assay showing that BnaA06.SnRK2 reduced the stability of BnaC04.bZIP16. Equal amounts of protein (30 μg) extracted from 15‐DAF‐old WT and *OE‐BnaA06.SnRK2* seeds were incubated with 100 ng of recombinant His‐BnaC04.bZIP16, His‐BnaC04.bZIP16^S151A^ and His‐BnaC04.bZIP16^S151D^ protein at 25°C and samples were taken at 6 time points from 0 to 80 min. The amount of protein remaining was measured using anti‐His antibodies, and immunoblot results were quantified using ImageJ software. (d) The relative amount of protein remaining was calculated according to (c) and a semilogarithmic decay curve was plotted. His‐BnaC04.bZIP16, His‐BnaC04.bZIP16^S151A^ and His‐BnaC04.bZIP16^S151D^ were set to 1 when 0 min was used as the correction parameter in the WT background. (e) Subcellular localization of BnaC04.bZIP16 with mutation of the Ser at position 151. S151D, the serine at position 151 is mutated to aspartic acid.

Given the kinase characteristics of BnaA06.SnRK2, the kinase‐substrate relationship between BnaA06.SnRK2 and BnaC04.bZIP16 was determined by an *in vitro* kinase assay. To investigate whether Ser151 detected by LC–MS/MS is the key site of BnaA06.SnRK2‐mediated phosphorylation, we used an equivalent amount of BnaC04.bZIP16^S151A^ protein as the substrate for the kinase assay, in which Ser151 was replaced with alanine. The phosphorylation intensity of BnaC04.bZIP16^S151A^ protein was significantly lower than that of native BnaC04.bZIP16 protein, indicating that Ser151 was a key site of BnaA06.SnRK2‐mediated phosphorylation of BnaC04.bZIP16. Interestingly, we detected two protein bands with anti‐pSer antibodies. According to the molecular weight of the protein, it was estimated that the upper band was the self‐phosphorylated band of GST‐BnaA06.SnRK2 (~ 65 kDa), and the lower band was the band of His‐BnaC04.bZIP16 phosphorylated by BnaA06.SnRK2 (~ 50 kDa) (Figure 6a). Next, the EMSA assay was performed using harboring protein by *in vitro* kinase assay. The result showed that with the increase in GST‐BnaA06.SnRK2 protein, the phosphorylation intensity of BnaC04.bZIP16 protein increased, but the binding strength of BnaC04.bZIP16 protein to the biotin‐labeled *BnaA08.OLEOSIN1* promoter probe decreased (Figure 6b). The results suggested that BnaA06.SnRK2‐mediated phosphorylation of BnaC04.bZIP16 attenuates binding to the promoters of target genes *in vitro*.

On the other hand, protein phosphorylation is often associated with the stability of the target proteins (Wang et al., 2020). Therefore, we attempted to examine the effect of BnaA06.SnRK2‐mediated phosphorylation on the stability of BnaC04.bZIP16 protein by a cell‐free protein degradation assay. The results showed that the half‐life of His‐BnaC04.bZIP16 protein was significantly degraded to 10 min when the protein extracts of 15 DAF seeds of *OE‐BnaA06.SnRK2* were used, while the half‐life of BnaC04.bZIP16 protein was 20 min when incubated with that of the wild‐type. Furthermore, using BnaC04.bZIP16^S151D^ protein as a substrate, the protein half‐life was reduced to 10 min when incubated with protein extracts of the wild‐type. We also found that the protein half‐life of His‐BnaC04.bZIP16^S151A^ protein was almost the same when incubated with wild‐type or *OE‐BnaA06.SnRK2* protein extracts. This further confirmed that Ser151 is a key phosphorylation site of BnaA06.SnRK2‐mediated phosphorylation of BnaC04.bZIP16 protein. The addition of MG132, a 26S proteasome inhibitor, notably delayed the degradation of His‐BnaC04.bZIP16 protein, indicating that the protein is under the ubiquitin/26S proteasome degradation pathway (Figure 6c,d).

In addition, by transient expression of BnaC04.bZIP16^S151D^‐eGFP in tobacco cells, in which Ser151 is replaced with aspartic acid to mimic the continuous phosphorylation status of BnaC04.bZIP16 protein, we found that BnaC04.bZIP16^S151D^‐GFP is located in both the nucleus and cytoplasm, unlike native BnaC04.bZIP16, which is located only in the nucleus (Figure 6e).

Altogether, these results strongly indicate that BnaA06.SnRK2‐mediated phosphorylation of BnaC04.bZIP16 weakens the affinity of BnaC04.bZIP16 for its target gene promoters and reduces its stability.

### Overexpression of *BnaA06.SnRK2* changed the level of *BnaA08.OLEOSIN1* and *BnaC03.PDH‐E1 BETA* transcription without altering the expression of *BnaC04.bZIP16*

To further explain the effect of BnaA06.SnRK2‐mediated phosphorylation of BnaC04.bZIP16 on fatty acid content in *B. napus* seeds, two transgenic lines overexpressing *BnaA06.SnRK2* were generated. The RT‐qPCR results showed that overexpression of *BnaA06.SnRK2* did not change the transcription level of *BnaC04.bZIP16* but significantly increased the expression levels of both *BnaA08.OLEOSIN1* and *BnaC03.PDH‐E1 BETA* (Figure 5a). Consistent with our previous views, BnaA06.SnRK2‐mediated phosphorylation of BnaC04.bZIP16 protein weakens the binding strength to the *BnaA08.OLEOSIN1* and *BnaC03.PDH‐E1 BETA* promoters, which may lead to changes in the transcription levels of *BnaA08.OLEOSIN1* and *BnaC03.PDH‐E1 BETA*.

**Figure 7:**
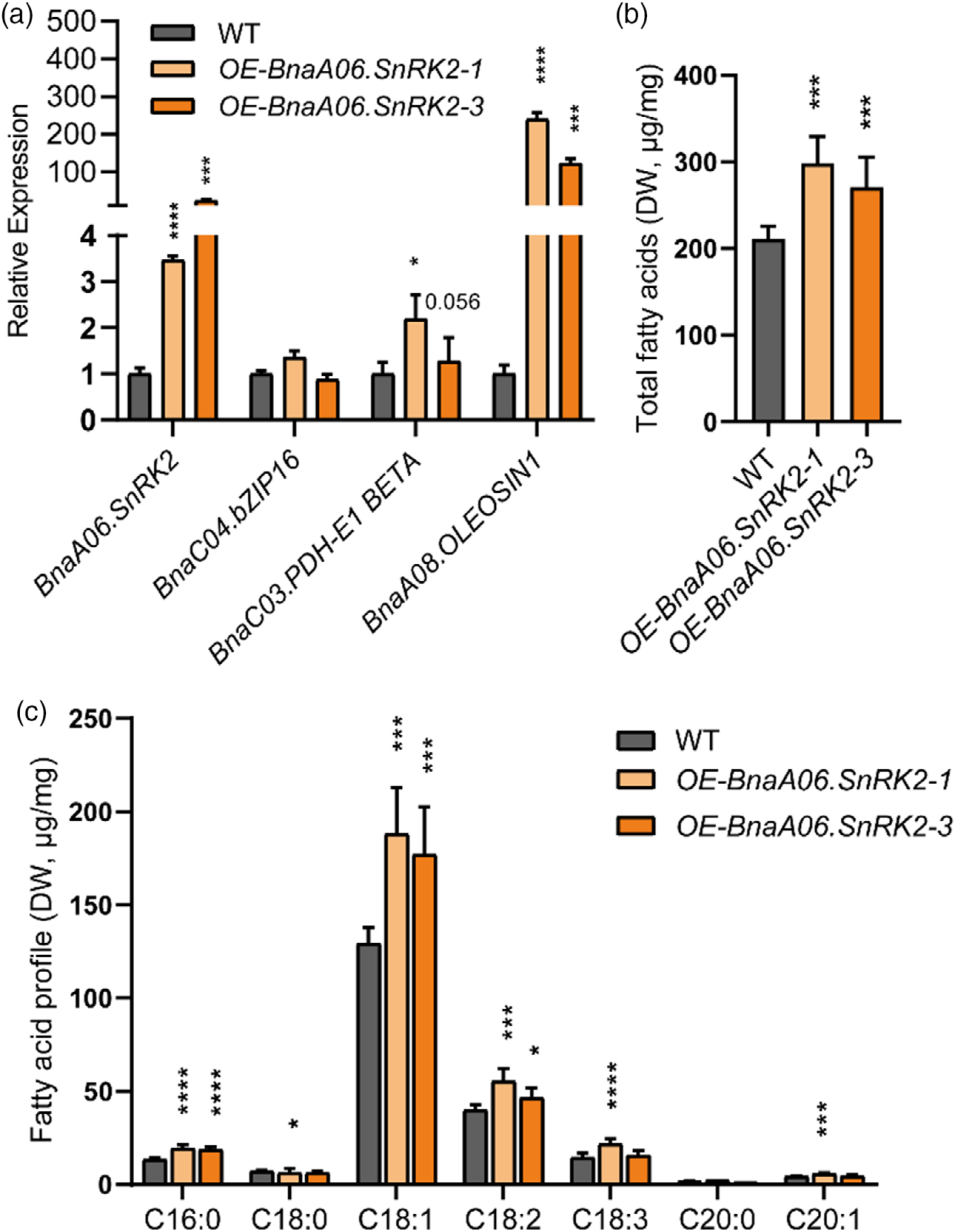
*BnaA06.SnRK2* alters the expression of *BnaA08.OLEOSIN1* and *BnaC03.PDH‐E1 BETA*. (a) Relative expression levels of *BnaC04.bZIP16*, *BnaA08.OLEOSIN1* and *BnaC03.PDH‐E1 BETA* in wild‐type and *OE‐BnaA06.SnRK2* seeds. Error bars indicate SD (*n* = 3). (b, c) Total fatty acid content and fatty acid profile in *BnaA06.SnRK2*‐transgenic *B. napus* seeds. Error bars indicate SD (*n* = 5). Asterisks indicate significant differences compared to the control wild type (****P < 0.001; ***P < 0.005; **P < 0.05; *P < 0.01. Student's *t* test).

Accordingly, the total fatty acid content of *OE‐BnaA06.SnRK2* seeds was significantly increased by at least 20% compared with that of wild‐type seeds in *B. napus* (Figure 7b), and the content of the main fatty acid compositions, especially oleic acid (C18:1), in *OE‐BnaA06.SnRK2* seeds was also significantly increased (Figure 7c).

## DISCUSSION

Improvement in the seed oil content of *B. napus* is a critical breeding objective. A deep understanding of the mechanism underlying FA synthesis and accumulation in *B. napus* is beneficial for this objective. However, to date, the understanding is still very limited, even though a rough regulatory network of fatty acid metabolism has been established in *Arabidopsis* (Su et al., 2021). In this study, we characterized the function of *BnaC04.bZIP16*, a member of the G subfamily of bZIP TFs, and uncovered its role as a negative regulator of seed FA accumulation in *B. napus*.

In *Arabidopsis*, the bZIP family has crucial functions. bZIP can respond to light signals (Xing et al., 2022), endoplasmic reticulum stress (Iwata et al., 2008), metal ion stress (Farinati et al., 2010), abscisic acid (ABA) (Yang et al., 2017), and various abiotic stresses (Bi et al., 2021). There are 13 subgroups in the bZIP family, including A–K, M, and S (Droge‐Laser et al., 2018), in which the genes reported to be involved in fatty acid metabolism are mainly members of subfamily A. *BnaC04.bZIP16*, a member of the G subfamily of the bZIP family, is highly expressed in siliques and seeds, suggesting its functional relevance during seed development. Functional studies using overexpression and knockout lines demonstrated that *BnaC04.bZIP16* represses the expression of key FA biosynthetic genes, including *BnaA08.OLEOSIN1* and *BnaC03.PDH‐E1 BETA*, by directly binding to G‐box motifs in their promoters. This establishes *BnaC04.bZIP16* as a direct transcriptional repressor of FA biosynthesis in *B. napus* seeds. Interestingly, we observed opposite expression patterns of *BnaC04.bZIP16* in siliques and seeds, suggesting a possible source–sink regulatory mechanism (Li, Song, et al., 2018; Liu et al., 2012). The extent to which silique wall expression contributes to seed FA levels in transgenic lines remains an open question for future investigation.

Transcription factors may perform distinct roles across different biological processes, functioning either as negative or positive regulators depending on the specific context. For example, GBF1 acts as a negative regulator of hypocotyl elongation under blue light, while serving as a positive regulator of cotyledon expansion and lateral root development during photomorphogenesis (Mallappa et al., 2006). Similarly, bZIP68 has been reported to repress stress‐responsive genes while promoting growth‐related gene expression; its loss‐of‐function enhances stress tolerance but inhibits growth (Li et al., 2019). OsAREB1 (also known as OsABI5 or bZIP39) upregulates ABA/stress‐related genes such as *RD29A* and *RD29B*, while downregulating flowering‐related genes including *FT*, *SOC1*, *LFY*, and *AP1*, which play opposing roles in stress response and flowering regulation (Jin et al., 2010). It is possible that BnaC04.bZIP16 also exhibits such dual regulatory roles, as suggested by our dual‐luciferase assay results (Figure S5b), though further investigation is needed to confirm this. In our study, while several candidate promoters showed modest activation by BnaC04.bZIP16, the strongest and most reproducible effects were observed for repressed targets such as *BnaA08.OLEOSIN1* and *BnaC03.PDH‐E1 BETA*, which are directly involved in fatty acid biosynthesis and storage. Therefore, we prioritized these genes for detailed functional validation. In contrast, positively affected candidates and degradation‐related genes such as *BnaC02.PKT1_KAT5* were not further investigated in the present work, but they represent promising targets for future studies on fatty acid catabolism.

Post‐translational regulation is a key mechanism by which bZIP TFs are modulated, particularly through phosphorylation. Several studies have shown that SnRK kinases participate in FA metabolism by regulating transcription factors such as WRI1 and DGAT1 (Caldo et al., 2018; Zhai et al., 2017). We demonstrated that BnaA06.SnRK2 directly phosphorylates BnaC04.bZIP16, which in turn reduces its stability and DNA‐binding activity. Overexpression of *BnaA06.SnRK2* led to increased seed FA content, likely by attenuating the repressive effect of BnaC04.bZIP16 on target genes. This suggests that *SnRK2* promotes FA biosynthesis, potentially via both direct and indirect mechanisms. Additionally, *SnRK2* is known to mediate responses to abiotic stress (Hasan et al., 2022), and lipid remodeling is often associated with stress adaptation (Liang et al., 2019). Our results raise the possibility that *SnRK2*‐dependent modulation of FA biosynthesis may be integrated with stress signaling pathways. We also observed upregulation of *BnaA06.SnRK2* in OE‐*BnaC04.bZIP16* lines, suggesting the existence of a possible feedback loop, though whether this regulation is direct or indirect remains to be clarified. Furthermore, the potential involvement of BnaC04.bZIP16 in the regulation of fatty acid desaturation, such as through modulation of *Bna.FAD2* gene, remains to be explored in future studies. Investigating whether BnaC04.bZIP16 affects the balance between saturated and unsaturated fatty acids will provide a more comprehensive understanding of its role in lipid metabolism.

To validate our findings in a broader genetic background, future studies will test the function of BnaC04.bZIP16 in elite *B. napus* cultivars with higher seed oil content. In this study, we selected Westar primarily due to its high transformation efficiency and well‐characterized genome, which facilitates gene functional analysis. However, we acknowledge that Westar has relatively low seed oil content (~41%) compared to most commercial cultivars in China (>45%). To assess the breeding potential of the identified regulatory module, future functional validation will be performed in high‐oil‐content cultivars such as Zhongshuang11.

**Figure 8:**
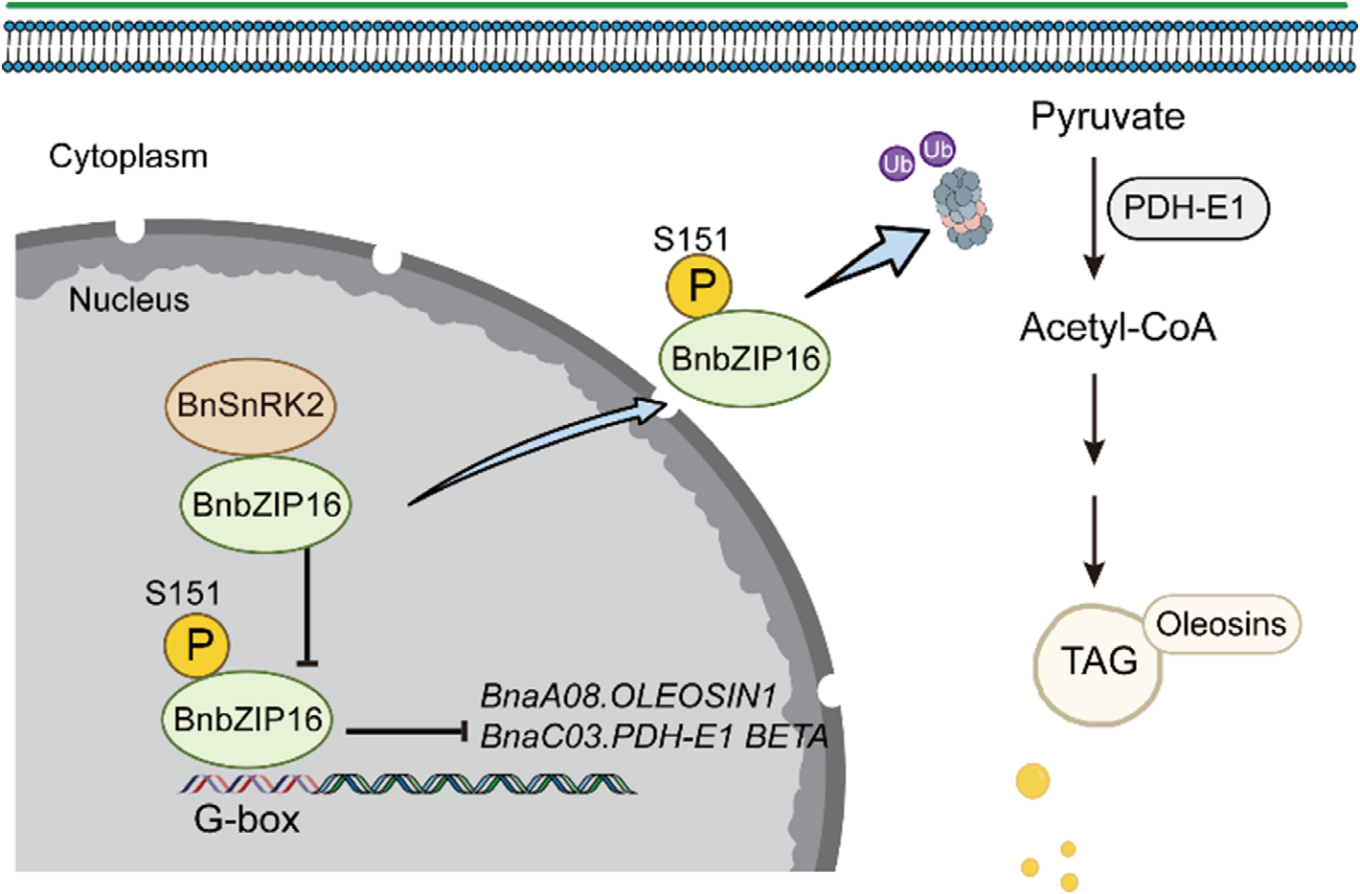
Model of the role of *BnaC04.bZIP16* in regulating fatty acid metabolism in *B. napus*. By directly inhibiting the expression of *BnaA08.OLEOSIN1* and *BnaC05.PDH‐E1 BETA*, BnaC04.bZIP16 negatively regulates the fatty acid metabolism pathway and reduces fatty acid content in *B. napus* and *Arabidopsis* seeds. BnaC04.bZIP16 can be phosphorylated by BnaA06.SnRK2, and phosphorylation reduces protein stability and weakens the binding strength of BnaC04.bZIP16 to *BnaA08.OLEOSIN1* and *BnaC03.PDH‐E1 BETA* promoters. In conclusion, *BnaC04.bZIP16* can negatively regulate the fatty acid content of seeds and can be used as a high‐quality genetic resource for *B. napus* genetic improvement.

According to our results, we proposed a working model for BnaC04.bZIP16 (Figure 8). BnaC04.bZIP16, as a transcription factor, directly regulates *BnaA08.OLEOSIN1* and *BnaC03.PDH‐E1 BETA*, thereby reducing the fatty acid content of seeds. The phosphorylation of BnaC04.bZIP16 mediated by BnaA06.SnRK2 decreases the ability of BnaC04.bZIP16 to regulate downstream target genes, thereby increasing seed fatty acid content. In summary, our study proposed a regulatory pathway, ‘BnaA06.SnRK‐BnaC04.bZIP16‐*BnaA08.OLEOSIN1/ BnaC03.PDH‐E1 BETA*’, with BnaC04.bZIP16 as the core for fatty acid biosynthesis, which provides a new perspective for the fatty acid biosynthesis regulatory network and is helpful for *B. napus* molecular breeding.

## MATERIALS AND METHODS

### Plant materials and growth conditions

The *A. thaliana* Columbia ecotype, *B. napus* cv. Westar, and *Nicotiana benthamiana* were used. *Arabidopsis* and *N. benthamiana* were grown in a greenhouse under 16 h of light and 8 h of darkness at 22°C. *B. napus* was grown in pots or experimental fields in Beijing, China, from March to July or from August to December. *Arabidopsis* and *B. napus* seedlings were placed at 4°C for 5 days and 1 month, respectively, before planting so that the initial growth state of each group was consistent.

### Identification of bZIP16 homologs in *B. napus*

To identify bZIP16 homologs in *B. napus*, the amino acid sequence of *A. thaliana* bZIP16 was used as a query to perform BLAST searches against the BnTIR database (https://yanglab.hzau.edu.cn/BnTIR/). Multiple sequence alignment was performed using ClustalW, and a phylogenetic tree was constructed using the Neighbor‐Joining method in MEGA 11 with 1000 bootstrap replicates.

### Vector construction and plant transformation

The constructs *OE‐BnaC04.bZIP16*, *OE‐BnaA06.SnRK2*, *OE‐BnaA08.OLEOSIN1*, and *OE‐BnaC03.PDH‐E1 BETA* were all constructed by Gateway recombination systems. The CDS of these genes, with the stop codon removed, was amplified and ligated to pHZM137 by the LR reaction, which included the 35S promoter, 3× FLAG tag, and the screening markers Basta and Hyg. The draft structure of the gene overexpression vector, taking pHZM137‐BnaC04.bZIP16 as an example, is shown in Figure S7a.

The CRISPR‐Cas9 mediated gene editing vector *KO‐BnaC04.bZIP16* was constructed by *Bsa*I digestion and T4 ligation. gDNA was designed on the CRISPR‐GE website (http://skl.scau.edu.cn/home/), and the amplification products obtained by PCR were ligated to pHSE401. The draft structure of the gene editing vector is shown in Figure S7b.

Both OE vectors and gene editing vectors were transformed into *Agrobacterium tumefaciens* GV3101.

Genetic transformation of *B. napus* cv. Westar was carried out by an *Agrobacterium*‐mediated hypocotyl transformation protocol as described previously (Dai et al., 2020). Westar was utilized as a transformation receptor. Genetic transformation of *A. thaliana* was carried out by the *Agrobacterium*‐mediated floral dip method (Clough & Bent, 2008). Columbia‐0 (Col‐0) was utilized as the transformation receptor.

### RT‐qPCR

For gene expression profiling, total RNA was extracted from various tissues of *B. napus* (Westar), including root, stem, leaf, bud, and seeds or siliques collected at 5, 10, 15, 20, 25, 30, 35, 40, and 54 days after flowering (DAF). To investigate the regulatory effect of BnaC04.bZIP16 on downstream target genes, siliques at 15 DAF were harvested from wild‐type (WT), overexpression (OE), and knockout (KO) lines. Total RNA was extracted using an RNA extraction kit (Tiangen, Beijing, China), and 3 μg of RNA was reverse‐transcribed using the One‐Step gDNA Removal and cDNA Synthesis Kit (Transgen, Beijing, China). The resulting cDNA was used as a template for qPCR with qPCR MasterMix (ABM, Richmond, BC, Canada).

### Subcellular localization

To examine the subcellular localization of some genes, such as BnaC04.bZIP16, the full‐length CDS was cloned into the plant expression vector pHZM27 to generate *35S::BnaC04.bZIP16‐GFP*. The construct was co‐infiltrated with the nuclear marker *35S::nucleus‐RFP* into 4‐week‐old *N. benthamiana* leaves using *Agrobacterium tumefaciens*. GFP and RFP signals were observed by confocal laser scanning microscopy.

### CUT&Tag

CUT&Tag was performed as previously described (Ouyang et al., 2021). Briefly, tissues harboring the *35S::BnaC04.bZIP16‐FLAG* construct were cross‐linked in 1% formaldehyde under vacuum, and the reaction was stopped using 2 M glycine. Then, the nuclei were extracted and resuspended with anti‐FLAG antibody (Sigma, Darmstadt, Germany) and secondary antibody (M21019M; Abmart, Shanghai, China). pG‐Tn5 (Vazyme, Nanjing, China) was added to the reaction solution, and then the DNA was segmented. Finally, the DNA was extracted and sequenced at BerryGenomics Company (Beijing, China).

### Yeast assays

We used the Matchmarker Gold Yeast Two‐hybrid System (Clontech, Mountain View, CA, USA) to perform yeast assays, which were performed as previously described (Hao et al., 2021). The CDS of *BnaA06.SnRK2*, with the stop codon removed, was ligated to pGBKT7. The CDS of *BnaC04.bZIP16*, with the stop codon removed, was ligated to pGADT7. The *BnaA08.OLEOSIN1* and *BnaC03.PDH‐E1 BETA* promoters (1.5 kb upstream of ATG) were ligated to linearized pHZM59 by the LR reaction. The draft structures of pGADT7‐BnaC04.bZIP16, pGBK‐BnaA06.SnRK2, and pHZM59‐BnaA08.OLEOSIN1 are shown in Figure S7c–e. The sequences of the *BnaA08.OLEOSIN1* and *BnaC03.PDH‐E1 BETA* promoters are listed in Table S1.

### Luciferase transient transactivation activity assay (dual‐luciferase assay)

The promoters of *BnaA08.OLEOSIN1* and *BnaC03.PDH‐E1 BETA* (1.5 kb upstream of ATG) were ligated to pHZM188 by the LR reaction as reporters (Figure S7f), including firefly luciferase (fLUC) and Renilla luciferase (rLUC), while *BnaC04.bZIP16* was used as the effector. Empty pHZM137 was used as a negative control. The reporters and effector were coinjected into tobacco leaves; the luciferase activity was quantified using a dual‐luciferase reporter assay system kit (Promega, Madison, WI, USA) by GloMax 2000 luminometer, and the relative promoter activity is expressed as 1000× fLUC/rLUC.

### Electrophoretic mobility shift assay (EMSA)

The recombinant protein His‐BnaC04.bZIP16 was purified from Transetta (DE3) chemical component cells (TransGen, Beijing, China). Biotin‐labeled probes designed according to the G‐box in *BnaA08.OLEOSIN1* and *BnaC03.PDH‐E1 BETA* promoters were synthesized by Sangon Company (Shanghai, China). EMSA was performed using an EMSA kit (Thermo, Waltham, MA, USA) with minor modifications. Briefly, equally purified recombinant proteins were preincubated with EMSA binding buffer at room temperature for 10 min, and then 20 fmol of biotin‐labeled probes with or without competing probes were added. Next, 5× loading buffer was added to stop the reaction. The incubated mixture was separated in a 6% native‐PAGE gel (0.5× TBE, 2.5% glycerol, 6% polyacrylamide) and transferred to a nylon film (Invitrogen, Carlsbad, CA, USA). Biotin‐labeled probes were detected by chemiluminescence and photographed with a Tanon 3500.

### Chromatin immunoprecipitation‐quantitative PCR (ChIP‐qPCR)

ChIP assays were performed as previously described with minor modifications (Li, Tian, et al., 2018; Liu et al., 2018). *B. napus* siliques harboring the *35S::BnaC04.bZIP16‐FLAG* construct and *35S::FLAG* construct were cross‐linked in 1% formaldehyde under vacuum, and the reaction was stopped using 2 M glycine. Nuclei were immunoprecipitated using anti‐FLAG antibody (Sigma, Darmstadt, Germany), and DNA was purified using a PCR purification kit (Qiagen, Beijing, China). Next, real‐time PCR was performed for quantitative analysis, and the amount of precipitated DNA is expressed as %Input, in which %Input = 2^ΔCt^ × 100%, where ΔCt = Ct (input) − Ct (IP).

### Split‐luciferase complementation and biomolecular fluorescence complementation assay

BiFC assay was performed in *N. benthamiana* leaves as described previously (Meng et al., 2023). The CDS of *BnaC04.bZIP16*, with the stop codon removed, was ligated to pEARLYGATE201‐nYFP (Figure S7g) by the LR reaction, and the CDS of *BnaA06.SnRK2*, with the stop codon removed, was cloned and inserted into pEARLYGATE202‐cYFP (Figure S7h) by the LR reaction. After the vector was expressed in tobacco leaves for 48 h, the YFP signals were observed under a confocal laser scanning microscope (Leica TCS SP5; Leica, Wetzlar, Germany).

Split‐luciferase complementation assay was performed by co‐infiltrating with *cLUC‐BnaC04.bZIP16* and *nLUC‐BnaA06.SnRK2* constructs in *N. benthamiana* leaves for 48 h. Then, the reactive substrate was added, and the CCD imaging system was used to detect the reaction.

### Pollen viability detection

In the morning, fresh flowers were collected, and the petals and pistils were carefully removed. The pollen material was then placed on a slide, treated with 2–3 drops of Alexander's staining solution (Coolaber, China), covered with a cover slip, and stained for 5–10 h before being observed under a microscope.

### *In vitro* phosphorylation assays

The recombinant proteins His‐BnaC04.bZIP16, His‐BnaC04.bZIP16^S151A^, and GST‐BnaA06.SnRK2 were expressed in *Transetta* (DE3) chemical component cells (TransGen, Beijing, China) and purified using Ni‐beads and GST‐beads (Smart Life Sciences, Beijing, China). The reaction system consisted of kinase protein (2 μg), substrate protein (3 μg), protein kinase reaction buffer (25 mm Tris–HCl, pH 7.4, 10 mm MgCl_2_, 1 mm DTT, 1 mm ATP), and GST buffer (25 mm Tris–HCl, pH 7.4, 100 mm NaCl, 10 mm MgCl_2_, 10% glycerol) and was incubated at 30°C for 30 min. Then, 5× loading buffer was added to stop the reaction. Finally, anti‐pSer (Immune Chem, Burnaby, BC, Canada) antibody was used to determine the intensity of the phosphorylation reaction.

### *In vivo* phosphorylation assays

The assay was performed as described previously with minor modifications (Wang et al., 2020). *OE‐BnaC04.bZIP16* siliques were ground into a fine powder with liquid nitrogen and resuspended in protein extraction buffer (25 mm Tris–HCl, pH 7.4, 150 mm NaCl, 1 mm EDTA, 1% NP40, 5% glycerol, 10 μm MG132, 1 mm sodium vanadate, 1 mm PMSF, 1× Cocktail) and then centrifuged twice (12000× ***g*** for 10 min each time), and the obtained supernatant was incubated with anti‐FLAG magnetic beads at 4°C for 1 h. Next, the magnetic beads were washed at 4°C 5 times with washing buffer (50 mm Tris–HCl, pH 7.4, 150 mm NaCl, 0.1% NP40, 1 mm sodium vanadate, 1 mm PMSF, 1× Cocktail). Furthermore, the bound proteins were eluted with 40 μl of 1× PBS and 10 μl of 5× loading buffer, boiled for 5 min, and separated by 10% (v/v) SDS‐PAGE. Finally, anti‐pSer (Immune Chem, Burnaby, BC, Canada) and anti‐FLAG antibodies were used for detection.

### LC–MS/MS

To identify the phosphorylation site of BnaC04.bZIP16 and the proteins that interact with it, we performed LC–MS/MS assays in the Protein Center of the Institute of Biophysics, Chinese Academy of Sciences (Beijing, China). The binding protein samples obtained from the *in vivo* phosphorylation assay were divided into two parts, each dissolved in 10% SDS‐PAGE gel, and then subjected to LC–MS/MS. One part, where electrophoresis was immediately stopped when the loading buffer had moved 2 cm into the separating gel, was used to identify interacting proteins of BnaC04.bZIP16. The other that was stopped after the protein markers were completely separated on the gel was used to determine the phosphorylation sites of BnaC04.bZIP16.

### Cell‐free degradation assays

Equal amounts of *OE‐BnA06.SnRK* and wild‐type *B. napus* siliques were ground into fine powder in liquid nitrogen and then resuspended in degradation buffer (25 mm Tris–HCl, pH 7.4, 10 mm NaCl, 10 mm MgCl_2_, 1 mm DTT, 4 mm PMSF, 1 mm ATP). After centrifugation twice (12 000× ***g*** for 10 min each time), the supernatant was placed on ice for use. According to the experimental design, in each group of experiments, the extracted protein (200 μl) and purified recombinant protein (20 μg) were mixed and divided into 6 tubes (20 μl per tube), then incubated at 25°C at the same time. The reactions were performed at 6 different time points, and anti‐His antibodies were used for detection. The remaining protein was quantified using ImageJ software.

### Total fatty acid and fatty acid profile assay

Total fatty acid of seeds was extracted using the protocol described in previous studies (Liang et al., 2019), and the fatty acid content was measured as a ratio of total fatty acid content to seed dry weight. The increase percentages were measured as the increase ratio = (fatty acid content of sample—fatty acid content of control)/fatty acid content of control × 100. The extracted methylated fatty acid was analyzed by GC–MS with an Agilent 7890A (HP‐FFAP, 30 × 0.25 mm ID, 0.25 μm, Santa Clara, CA, USA) in the Protein Center of the Institute of Biophysics, Chinese Academy of Sciences (Beijing, China).

### Soluble sugar content and total protein assay of seeds

Mature *B. napus* seeds were dried at 50°C for 1 week. After taking 0.1 g of seed and adding 1 mL of distilled water to grind it into a homogenate, we determined the soluble sugar content using a plant soluble sugar content detection kit (Solarbio, Beijing, China). Next, the light absorption value at 620 nm was detected by a microplate reader. Finally, the soluble sugar content was calculated according to a standard curve.

The mature *B. napus* seeds were dried at 50°C for 1 week, weighed, and boiled at 380°C with H_2_SO_4_ and catalyst (Kejltec, Coventry, UK) for 1 h. Then, the total N content was determined by a FOSS‐Kjeltec TM‐8400. Finally, total protein content = total N content × 6.25.

## AUTHOR CONTRIBUTIONS

ZH, CF, and WH conceived the project and designed experiments. WH performed the experiments. ZH, CF, YC, XG, and WH analyzed the data and interpreted the results. SL, JD, QL, and YW revised the manuscript. WH wrote the manuscript.

## CONFLICT OF INTEREST

The authors declare no conflicts of interest.

## Supporting information


**Figure S1.** BnaC04.bZIP16 (BnaC04g09600D) belongs to subgroup G of the bZIP family.
**Figure S2.** Phenotype of *BnaC04.bZIP16* transgenic *B. napus* seeds.
**Figure S3.** Relative expression levels of *BnaC04.bZIP16* in *B. napus* seeds and siliques.
**Figure S4.** GO annotation of the genes corresponding to the promoter fragments enriched in CUT&Tag assay.
**Figure S5.** Bimolecular luciferase assay of candidate target genes of BnaC04.bZIP16.
**Figure S6.** Analysis of phosphorylation sites.
**Figure S7.** Diagram of constructs used in this study.

**Table S1.** Primers and probes in paper.

**Table S2.** Sequence in paper.

## Data Availability

The data that support the findings of this study are available in the supplementary material of this article.
