# Supplementary material for: BnaC04.bZIP16 can be phosphorylated and inhibited by BnaA06.SnRK2 and negatively regulates the accumulation of fatty acids in Brassica napus
Source: Plant J. 2025 Oct 3;124(1):e70506. doi: 10.1111/tpj.70506 (PMC12494420; doi:10.1111/tpj.70506)
Supplement: Supplementary file 1 — Figure S1. BnaC04.bZIP16 (BnaC04g09600D) belongs to subgroup G of the bZIP family. Figure S2. Phenotype of BnaC04.bZIP16 transgenic B. napus seeds. Figure S3. Relative expression levels of BnaC04.bZIP16 in B. napus seeds and siliques. Figure S4. GO annotation of the genes corresponding to the promoter fragments enriched in CUT&Tag assay. Figure S5. Bimolecular luciferase assay of candidate target genes of BnaC04.bZIP16. Figure S6. Analysis of phosphorylation sites. Figure S7. Diagram of constructs used in this study. [file TPJ-124-0-s003.docx]

**SUPPORTING INFORMATION**

Wei Hui^1,2^, Shuangshuang Li^1,2^, Jinhao Ding^1,2^, Qianru Li^1,2^, Yuhong Chen^1^, Yanhui Wang^3^, Xupeng Guo***** ^1^, Chengming Fan***** ^1^, Zanmin Hu***** ^1,2^

^1^ Key Laboratory of Seed Innovation, Institute of Genetics and Developmental Biology, Innovation Academy for Seed Design, Chinese Academy of Sciences, Beijing, 100101, China.

^2^ College of Advanced Agricultural Sciences, University of Chinese Academy of Sciences, Beijing, 100049, China.

^3^ Leshan Academy of Agricultural Sciences, Leshan, Sichuan, China

**
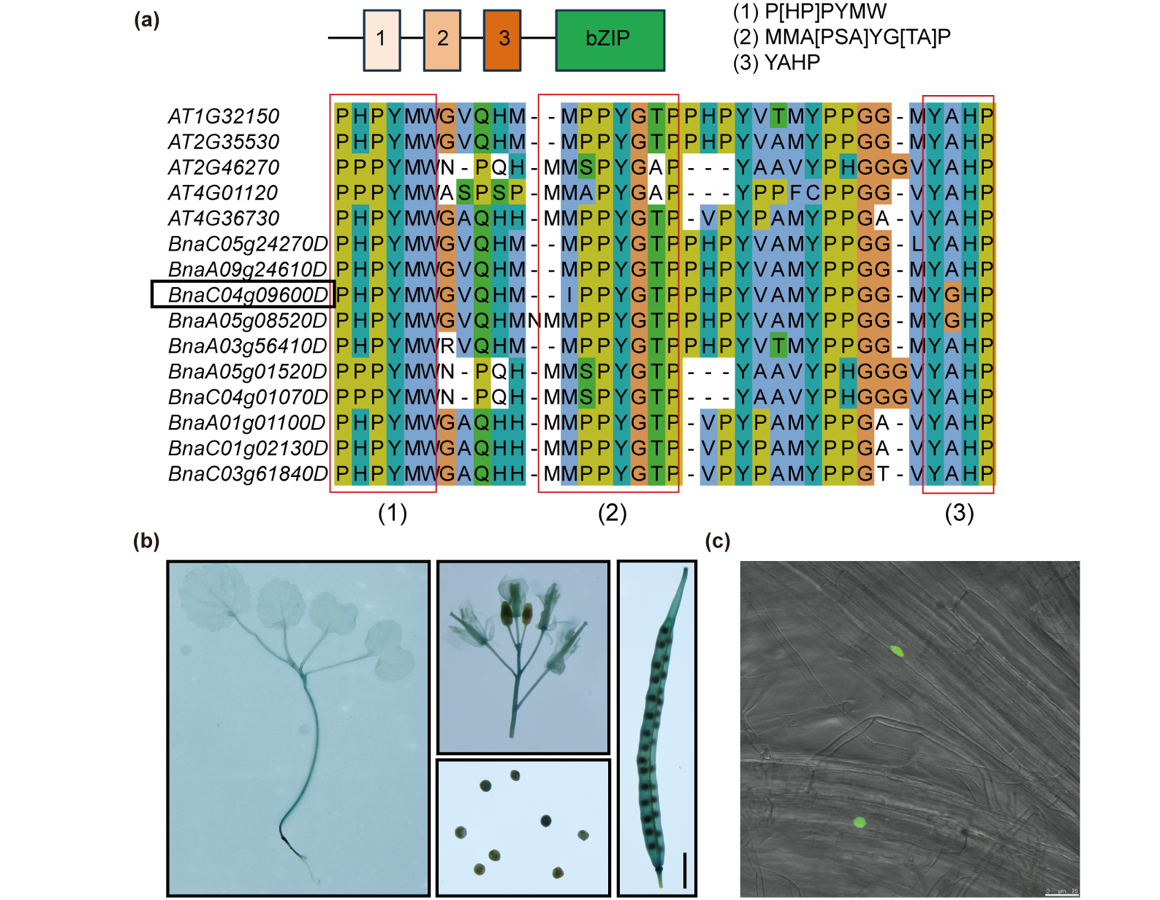
**

**Figure S1** BnaC04.bZIP16 (BnaC04g09600D) belongs to subgroup G of the bZIP family. (a) Sequence comparison of members of subfamily G of the bZIP family in *Arabidopsis* and *B. napus*. (b) The expression of *BnaC04.bZIP16* was detected in *proBnaC04.bZIP16::GUS* transgenic *B. napus*. β-glucuronidase (GUS) activity was detected in seedlings, flowers, siliques and 15 DAF seeds. Scale bars correspond to 1 cm. (c) Subcellular localization of BnaC04.bZIP16-eGFP in *Arabidopsis* roots. Bar, 25 μm.


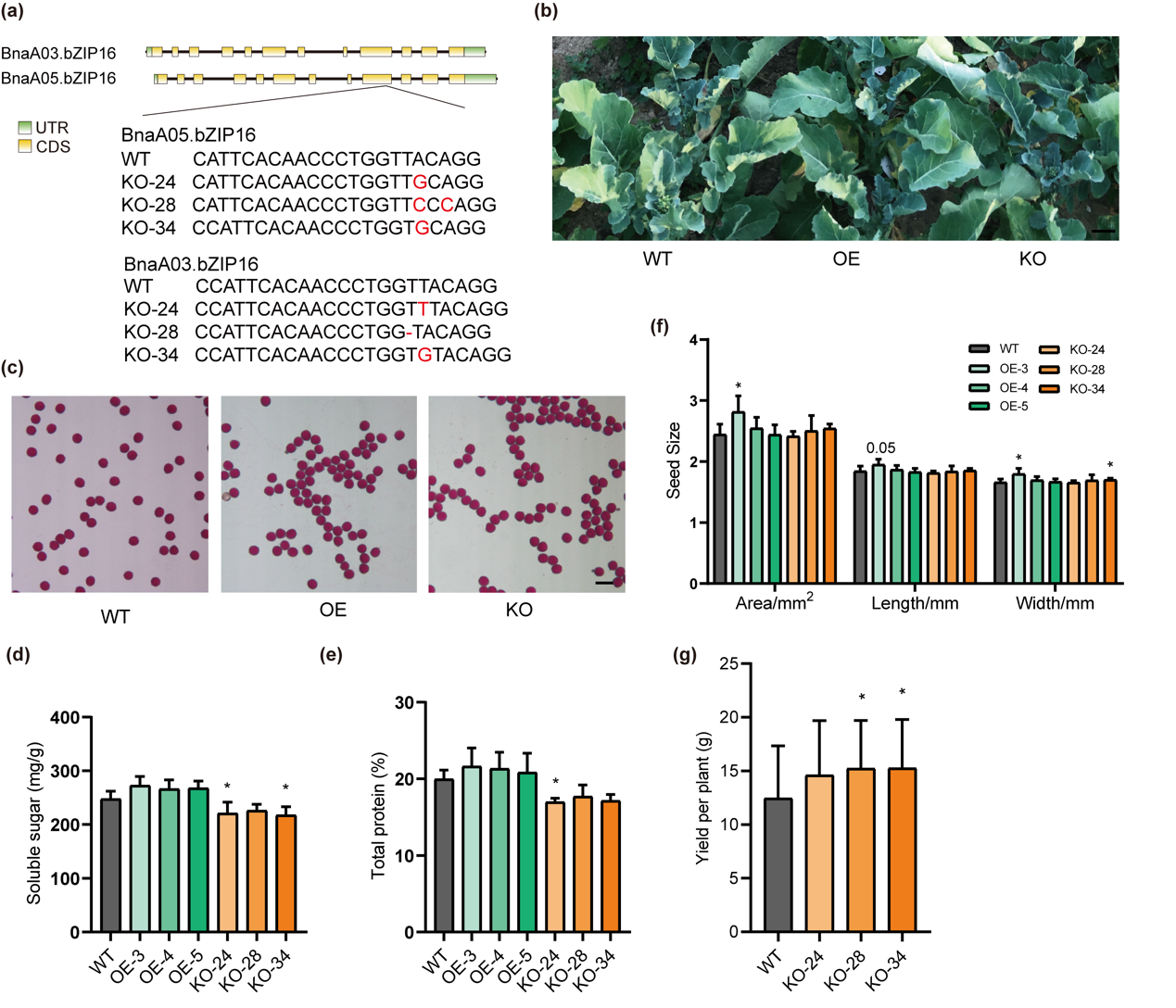
 **Figure S2** Phenotype of *BnaC04.bZIP16* transgenic *B. napus* seeds. (a) PCR sequencing results of KO-24, KO-28 and KO-34 mutant. (b) *B. napus* bud stage phenotype in *OE-BnaC04.bZIP16*, KO-mutant lines and WT. Scale bar, 1 cm. (c) Pollen staining of WT, *OE-BnaC04.bZIP16* and KO-mutant *B. napus*. Scale bar, 10 μm. (d-e) Content of soluble sugars and total protein in *OE-BnaC04.bZIP16*, KO-mutant lines and WT. (f-g) Seed size (length, width and area) and yield per plant in *OE-BnaC04.bZIP16*, KO-mutant lines and WT.


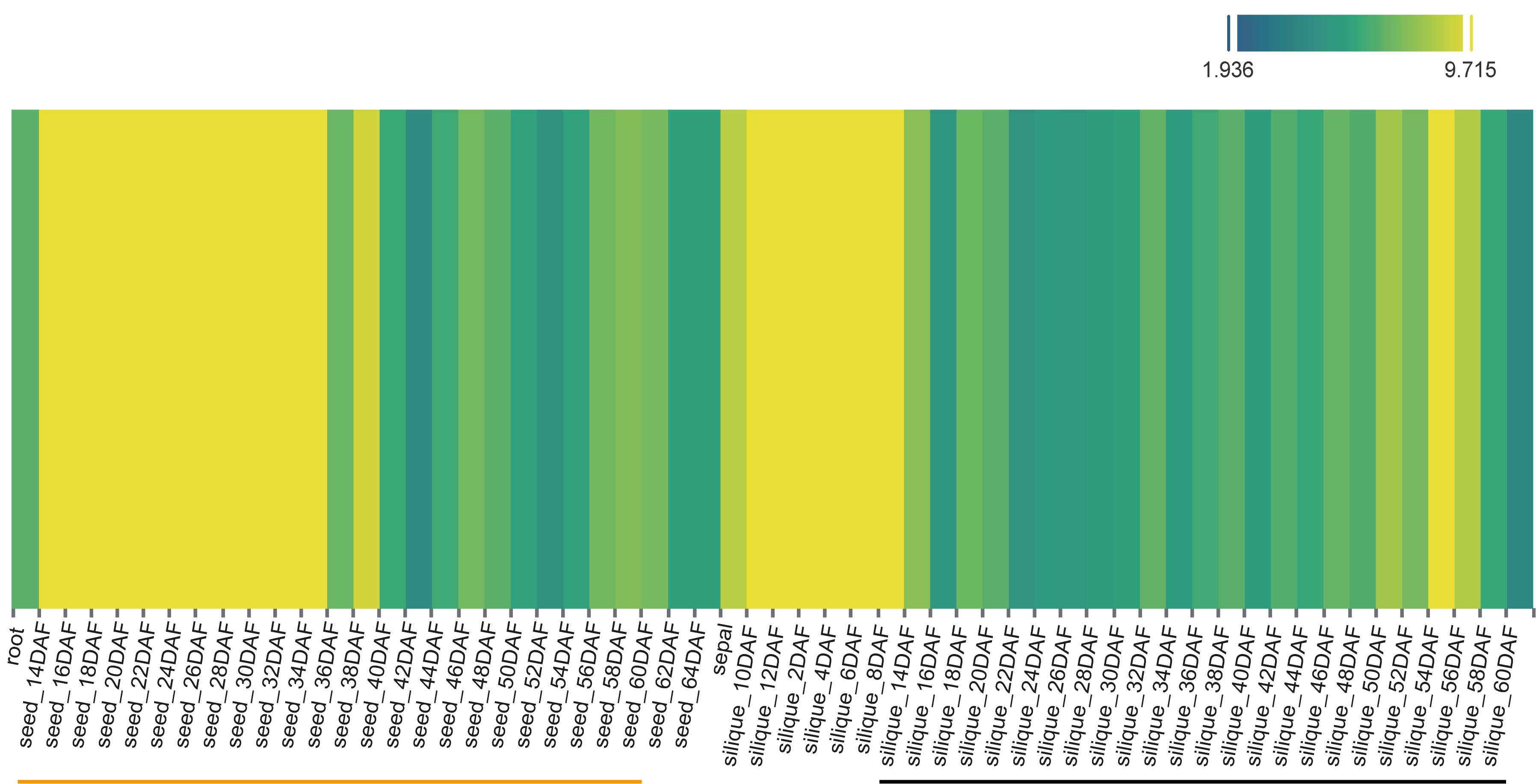


**Figure S3** Relative expression levels of *BnaC04.bZIP16* in *B. napus* seeds and siliques. Data were from BnTIR. The lines represent periods of opposite expression patterns in seeds (orange) and siliques (black).


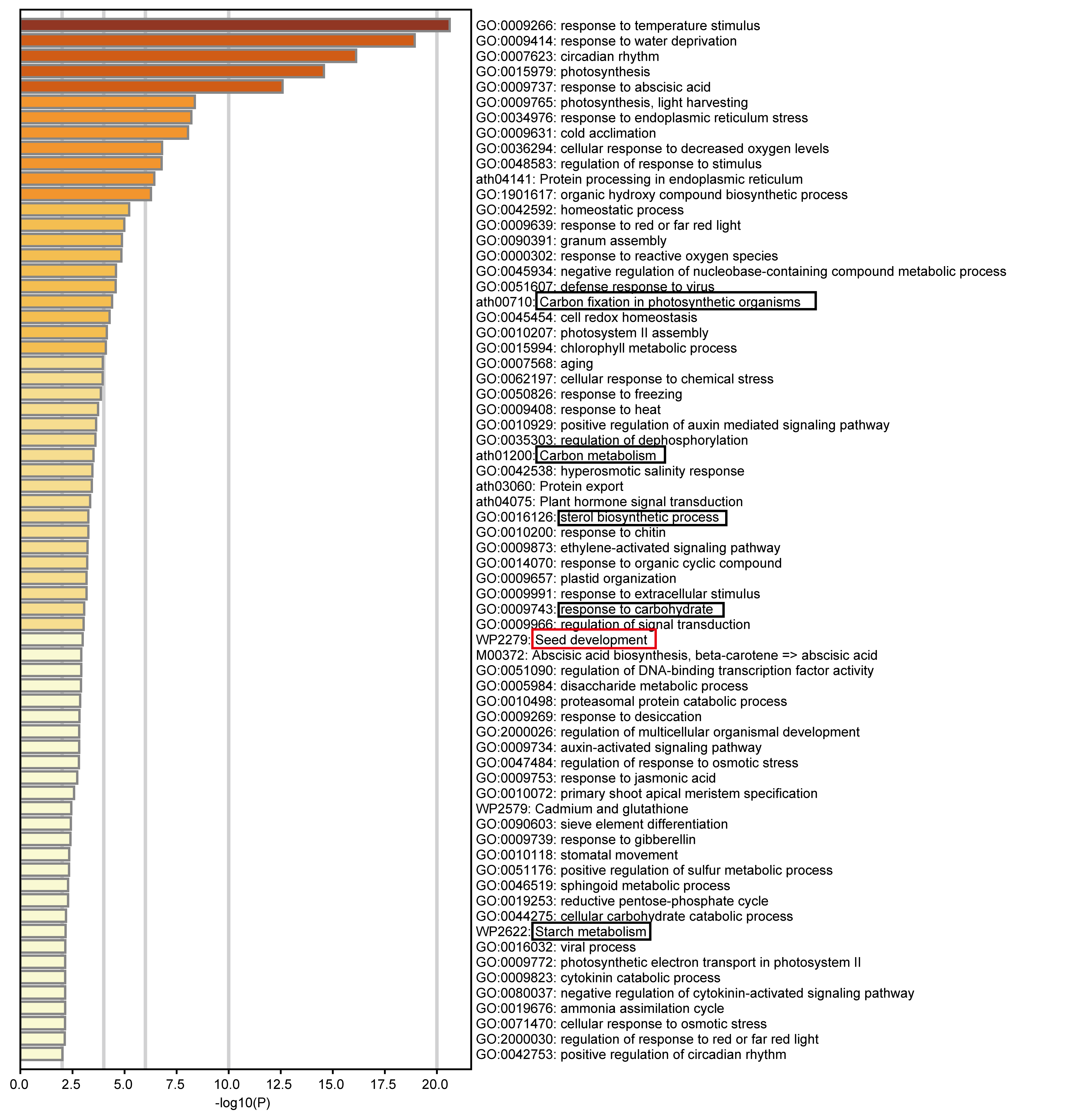


**Figure S4** GO annotation of the genes corresponding to the promoter fragments enriched in CUT&Tag assay. Go enrichment analysis was performed on Metascape (http://metascape.org/gp/).


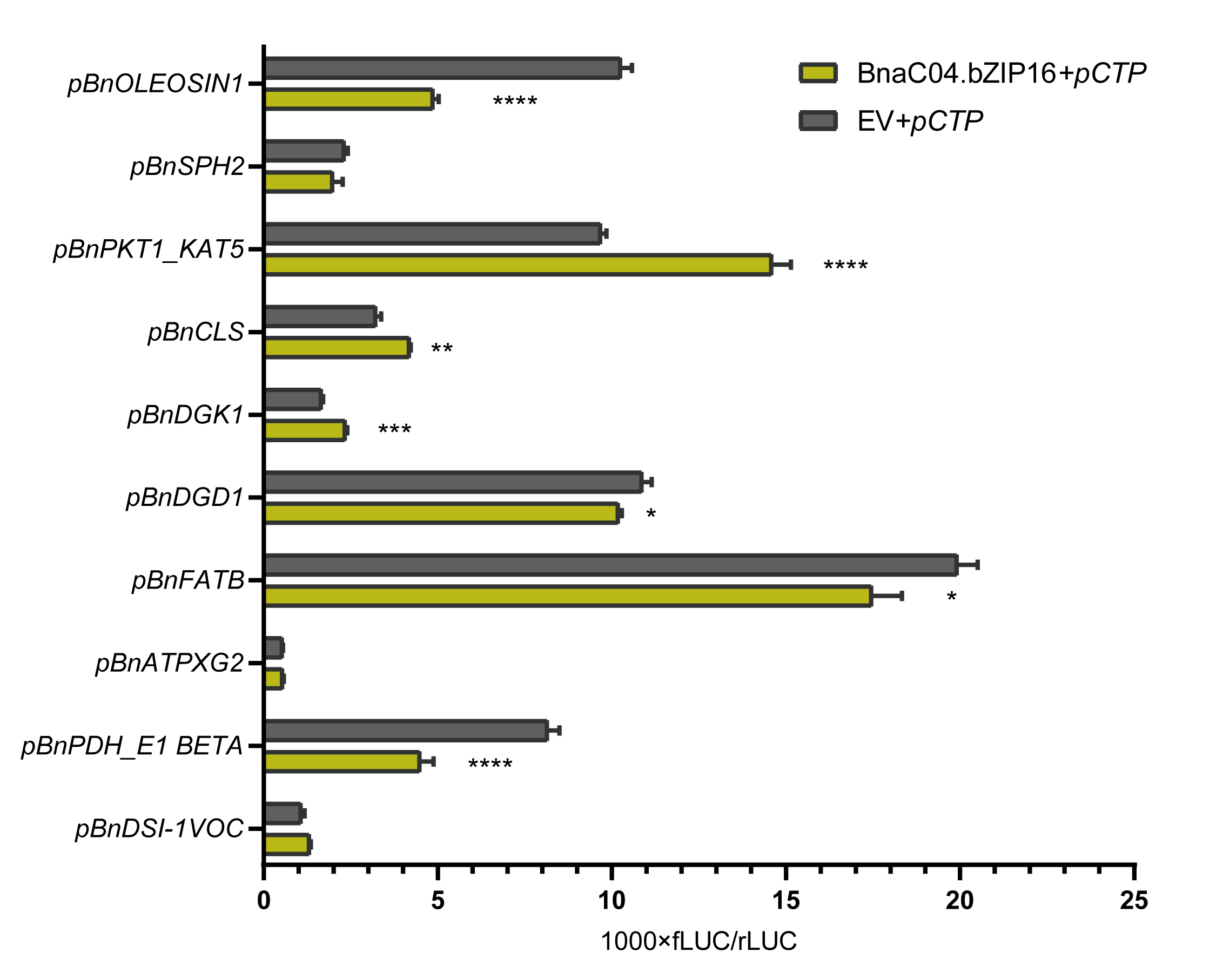


**Figure S5** Bimolecular luciferase assay. From the CUT&Tag results, 10 genes were selected according to the function of the genes, and then BnaC04.bZIP16 was subjected to bimolecular luciferase experiments with the promoters of these 10 genes. The 10 genes are *BnaA08.OLEOSIN1* (BnaA08g14540D), *BnaA08.SPH2* (BnaA08g09710D), *BnaC02.PKT1_KAT5* (BnaC02g38800D), *BnaC09.CLS* (BnaC09g22540D), *BnaC02.DGK1* (BnaC02g01400D), *BnaC05.DGD1* (BnaC05g41530D), *BnaA06.FATB* (BnaA06g04900D), *BnaC07.ATPXG2* (BnaC07g27360D), *BnaC03.PDH-E1 BETA* (BnaC03g59130D) and *BnaA06.DSI-1VOC* (BnaA06g04170D). pCTP, promoters of genes obtained by CUT&Tag assay. Asterisks indicates significant differences (****, *P* < 0.001; ***, *P* < 0.005; **, *P* < 0.01; *, *P* < 0.05. Student’s *t* test).


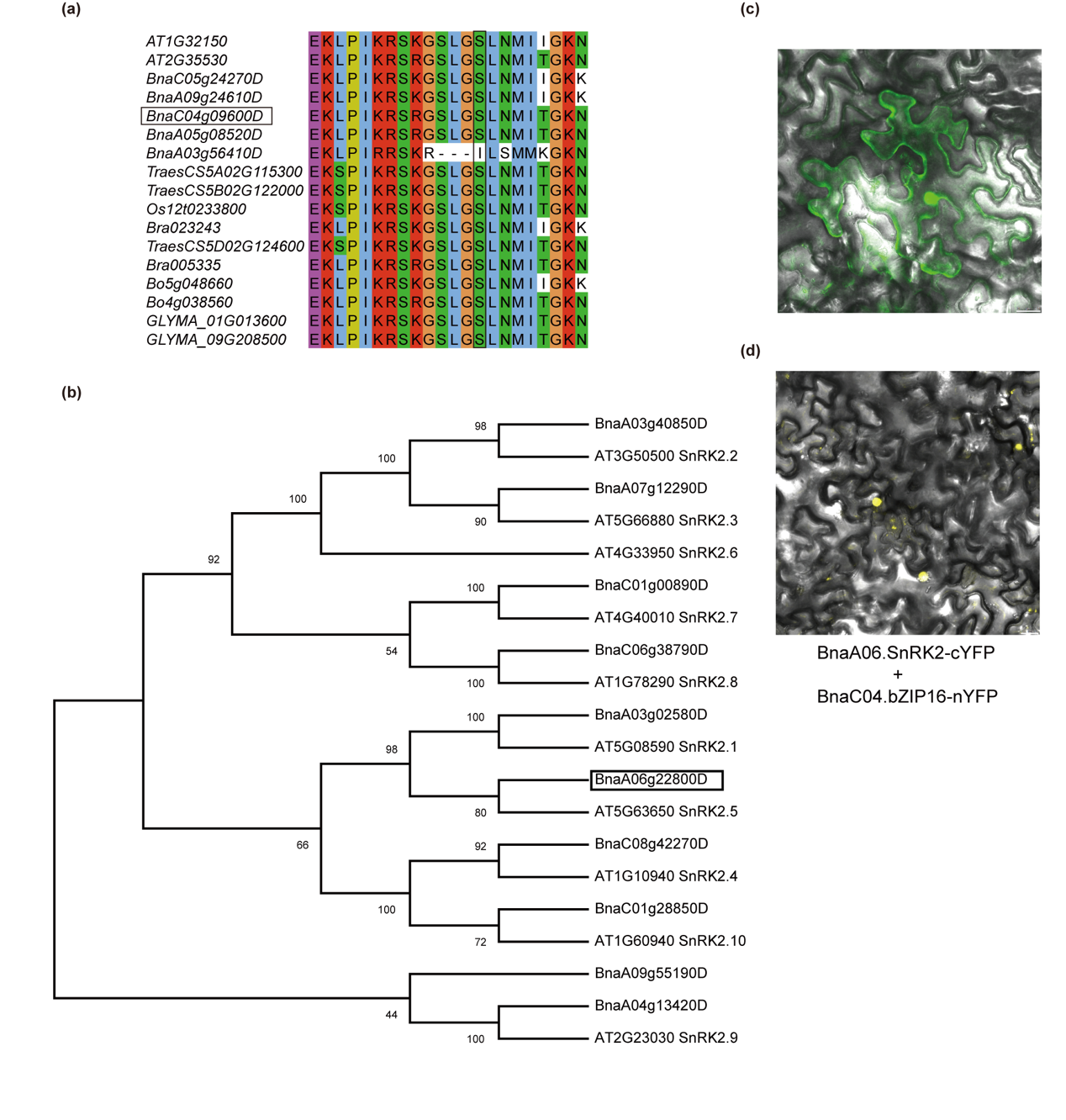


**Figure S6** Analysis of phosphorylation sites. (a) The phosphorylation site is highly conserved. Multiple sequence comparison of *BnaC04.bZIP16* and its homologues in *Arabidopsis thaliana*, *Brassica napus*, *Triticum aestivum*, *Oryza sativa*, *Brassica rapa*, *Brassica oleracea* and *Glycine max* showed that the phosphorylation sites were highly conserved (black box). (b) Phylogenetic tree of BnaA06.SnRK2. (c) Transient expression assay of *N. benthamiana* epidermal cells showing subcellular localization of BnaA06.SnRK2. GFP signals were observed using a confocal laser scanning microscopy. Scale bars, 25 μm. (d) BiFC analysis of the interaction between BnaC04.bZIP16 and BnaA06.SnRK2 in *N. benthamiana* leaf using Agrobacterium-mediated transient expression of the *BnaA06.SnRK2-cYFP* and *BnaC04.bZIP16-nYFP*. The YFP signal was visualized by a confocal microscope. Scale bars, 25 μm.


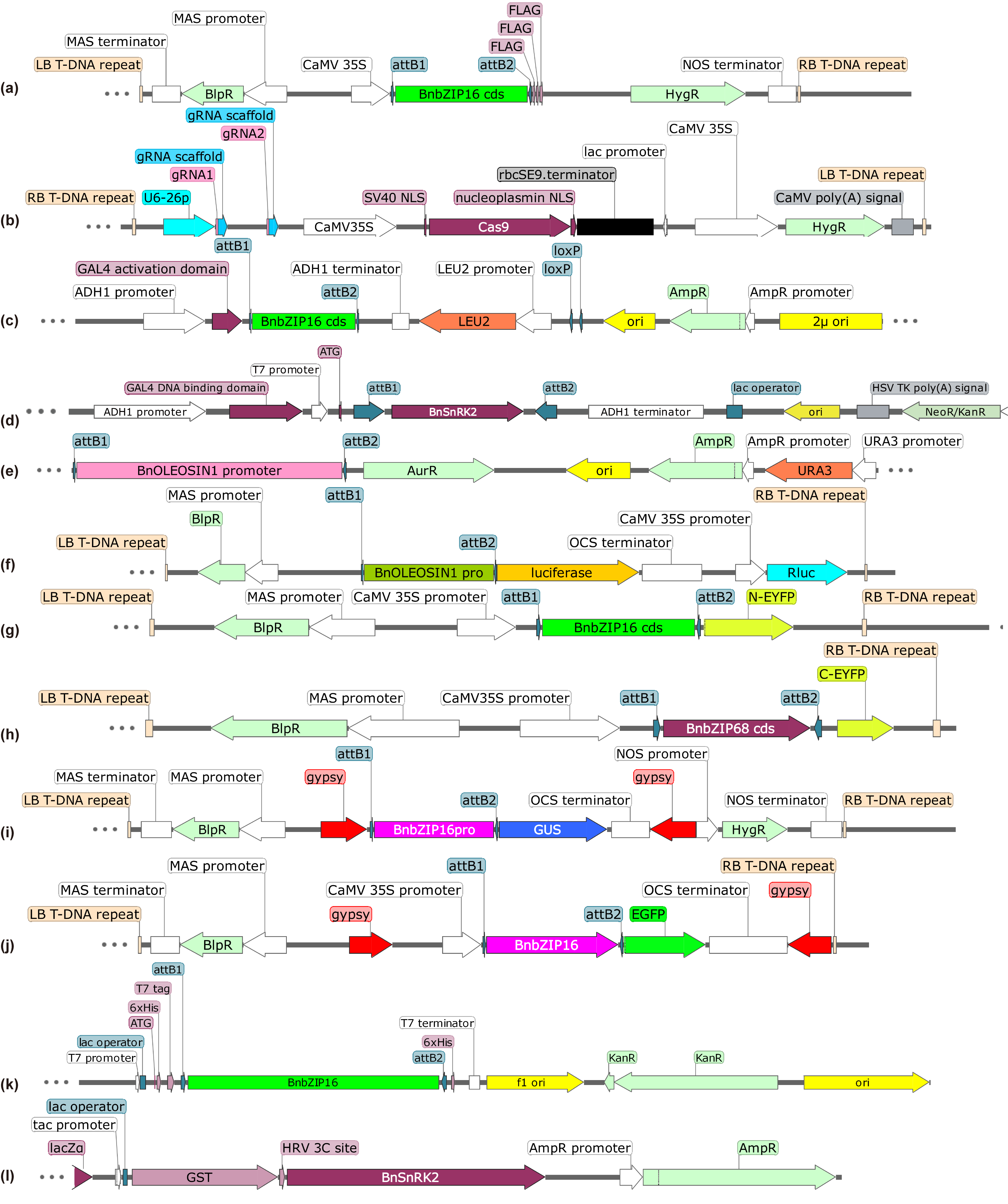
**Figure S7** Diagram of constructs used in this study. In this study, vectors used, except (b) and (l), are Gateway systems, and CDS with stop codons removed are used to construct them. (a) Diagram of the overexpression vector. (b) Diagram of the double target site-gene editing vector. (c-d) Diagrams of the prey vector (pGADT7) and bait vector (pGBKT7) in the yeast two-hybrid system. (e) Diagram of the pHZM59 vector in the yeast one-hybrid system. (f) Diagram of the reporter vector (pHZM188) used in the dual-luciferase assay. (g-h) Diagram of YFP fluorescent complementary vectors pEARLYGATE201-nYFP and pEARLYGATE202-cYFP used in the BiFC assay. (i) Diagrams of the expression vector pHZM108-GUS used in the GUS staining assay. (j) Diagrams of the expression vector pHZM27-eGFP used in subcellular localization. (k) Diagrams of the expression vector pET28a-His used in protein expression *in vitro*. (l) Diagrams of the expression vector pGEX-6P1-GST used for protein expression *in vitro*.
