# Supplementary material for: BnaC04.bZIP16 can be phosphorylated and inhibited by BnaA06.SnRK2 and negatively regulates the accumulation of fatty acids in Brassica napus
Source: Plant J. 2025 Oct 3;124(1):e70506. doi: 10.1111/tpj.70506 (PMC12494420; doi:10.1111/tpj.70506)
Supplement: Supplementary file 2 — Table S1. Primers and probes in paper. [file TPJ-124-0-s001.pdf]

Table S1 Primers and probes in paper

Prime name	Sequences (5' to 3')	Note
Primers for cloning the cDNAs and genomic DNAs		
BnbZIP16-F	ATGGCTAGCAATGAGATGGAG	BnaC04g09600D
BnbZIP16-R	CGTTGAGTTTTTGTAGGTATCATCAA	
BnbZIP68-F	ATGGGTAGTAGCGAGGTGGAC	BnaC05g24270D
BnbZIP68-R	GGCTGAGTTGTTGTAGGAACCAC	
BnbZIP16pro-F	TTAATCTCTAATAAACTCCGGCATTGCTCTT	BnaC04g09600D promoter
BnbZIP16pro-R	tatcaccagtgagaggtacctgcaaag	
BnPKT1_KAT5-F	ATGGAGAAAGCCACTGAAAGGCA	BnaC02g38800D
BnPKT1_KAT5-R	GTGACCGCTCCCATTAGTCTC	
BnOLEISIN1-F	ATGGCGGATACTGCTAGAACC	BnaA08g14540D
BnOLEISIN1-R	TGTAGTAGTGGTGGTGTGATGGG	
BnPDH_E1 BETA-F	ATGGCTGCGAGAATCCATG	BnaC03g59130D
BnPDH_E1 BETA-R	CTGGCAAAGCTGCTCAACG	
BnSnRK2-F	ATGGACAAGTATGAAGTTGTCAAGGAC	BnaA06g22800D
BnSnRK2-R	AGCTTTAGAAGACTCTTGGCTCTTATG	
BnbZIP16shortindel-F	TTATAATAAGCGTTCAGATCAGTTCCTTGCAT	
BnbZIP16shortindel-R	TACACTAAAAATCACTGCATGACAGTGTGC	
BnPKT1_KAT5pro-F	ctgactgttagcaccgccc	
BnPKT1_KAT5pro-R	cttttctcactgttaagcttaattcaagaaaaaaagg	
BnOLEISIN1pro-F	ctcaaaaagtgaactatatgagaaatgcaataagttagat	
BnOLEISIN1pro-R	tatgatcttgttgaagagagagatgagtttatgt	
BnPDH_E1 BETApro-F	ggtatatcgcggaaagttttgggc	
BnPDH_E1 BETApro-R	tttttttccccagggagagtgac	
Primes for constructing gene editing vectors		
KO-BnbZIP16-F	ATATATGGTCTCGATTGcattcacaaccctggttacGTTTTAGAGCTAGAAATAGC	vectors construction
KO-BnbZIP16-R	ATTATTGGTCTCGAAACGTAACCAGGGTTGTGAATGCAATCTCTTAGTCGACTCTAC	vectors construction
KO-BnOLEISIN-F	ATATATGGTCTCGATTGGACAGTGCAAGGATGAAGCGTTTTAGAGCTAGAAATAGC	vectors construction
KO-BnOLEISIN-R	ATTATTGGTCTCGAAACGtctcacccttgtcggaacCAATCTCTTAGTCGACTCTAC	vectors construction
KO-BnbZIP16-seqF	ggtgtggttgttgatcatttccttat	PCR and Sequencing
KO-BnbZIP16-seqR	tttgcttggttggccctagaaactt	PCR and Sequencing
KO-BnOLEISIN-seqF	ctggctaattgaacatagacgttcacttt	PCR and Sequencing
KO-BnOLEISIN-seqR	aacaccttattcctcatgaactagactcc	PCR and Sequencing
Primers for q-PCR		
qBnbZIP16-F	AATGAAGAAAACGCAAGCCTGA	
qBnbZIP16-R	TGGTTCTTGGTCGTCCTTGTC	
qBnbZIP68-F	ACGCAAGTCTTAGAGCAGAAAT	
qBnbZIP68-R	CGGCTCTTGCTCCTTGACAT	
qBnOLEOSIN1-F	AAGCTGCTACCGCAGTCAC	
qBnOLEOSIN1-R	GAAGTGCTACGGTGATGAGAGCC	
qBnPDH-E1 BETA-F	TGTCGACCTTTAATCCCAAGAA	
qBnPDH-E1 BETA-R	CCTCGAAAAGCAGTAGTTCATG	
qBnPKT1_KAT5-F	TGTACTGTTGCAAGAAGCTAGA	
qBnPKT1_KAT5-R	CAAGTCATCAACAGAGTCTCCT	
qBnSnRK2-F	GTTGGAGAAGCAAGGAATCCAGC	
qBnSnRK2-R	TTTGACATGCTTCTCATATTCATCCTCTTCTTC	
qBnACT7-F	TTCAATGTCCCTGCCATGTA	
qBnACT7-R	GAGACGGAGGATAGCGTGAG	
biotin-labeled probes		
embio-BnOLEOSIN1-F	CACCAACACGTGGATACTATTGCATGATGACGCCATTGACACATGACTCTC	
embio-BnOLEOSIN1-R	GAGAGTCATGTGTCAATGGCGTCATCATGCAATAGTATCCACGTGTTGGTG	
embio-BnPDH-E1 BETA-F	TACAAACACGTGTCTACTATAATCGTAAATGAACACGTGGCATAC	
embio-BnPDH-E1 BETA1-R	GTATGCCACGTGTTCATTTACGATTATAGTAGACACGTGTTTGTA	
embio-BnPKT1_KAT5-F	GTTCTTTTTTTTCCTACGTGGCTGGTTTGG	
embio-BnPKT1_KAT5-R	CCAAACCAGCCACGTAGGAAAAAAAAGAAC	
Primers for ChIP-qPCR		
ChIP-BnOLEOSIN1-P1-F	CGAGACAAAAACACAAACGTGTCCT	
ChIP-BnOLEOSIN1-P1-R	AGTTTATGTTTTGAAGTAGCTATGATTGTTGTAAATTGAT	
ChIP-BnPDH-E1 BETA-P1-F	aacccgtcctaactcctctacaag	
ChIP-BnPDH-E1 BETA-P1-R	aatctcctgtggcccccct	
ChIP-BnPKT1_KAT5-P1-F	atgacgttgttgccttctttcaact	
ChIP-BnPKT1_KAT5-P1-R	ccgtctctttttttcatgtttttctcccc	
ChIP-BnOLEOSIN1-P2-F	ctctcactctctctctcctttcatatcaac	Negative control
ChIP-BnOLEOSIN1-P2-R	ctgcttcttgaatcgtcgtaatgttctga	
Primers for site-mutation		
BnbZIP16[S151A]-F	GAGGAAGCTTGGGAGCTTTGAACATGATTACAGGAAAG	
BnbZIP16[S151A]-R	TTCAAAGCTCCCAAGCTTCCTCTTGATCTTTTGATAGG	
BnbZIP16[S151D]-F	GAGGAAGCTTGGGAgatTTGAACATGATTACAGGAAAG	
BnbZIP16[S151D]-R	GTTCAAatcTCCCAAGCTTCCTCTTGATCTTTTGATAGG	
Primers for detection vectors with Gateway system		
M13F	gtaaaacgacggccagt	pGWC
M13R	gtcatagctgtttcctg	
35S-F	CACTATCCTTCGCAAGACCCTTCC	pHZM137
137-seqR	GCGTCTCGCATATCTCATTAAAGCAG	
pGAD-GAL4.F	atgatgaagataccccaccaaaccc	pGADT7
pGADT7_2 R	gagatggtgcacgatgcacag	
PGBK-GAL4.F	GTGCGACATCATCATCGGAAG	pGBKT7
T7t_1R	AAACCCCTCAAGACCCGTTTAG	
AbAi.F	TCTTCGTTTCCTGCAGGTTT	pHZM59 sequencing
AbAi.R	ACATGGCAGTTTGGAGGTCT	
YHM59.F	CGAAGAGTAAAAAATTGTACTTGGCGG	pHZM59 linearization
YHM59.R	CGAAGACAGAAAATTTGCTGACATTGGTAATAC	
35S-F	CACTATCCTTCGCAAGACCCTTCC	pHZM188
Rluc.R	ATTTGCCTGATTTGCCCATA	
35S-F	CACTATCCTTCGCAAGACCCTTCC	pEARLYGATE201
C-EYFP-R	CTTCTCGTTGGGGTCTTTGCT	
35S-F	CACTATCCTTCGCAAGACCCTTCC	pEARLYGATE202
N-EYFP-R	TGGTCACGAGGGTGGGC	
35S-F	CACTATCCTTCGCAAGACCCTTCC	pHZM27
eGFP-R	CTCCTCGCCCTTGCTCA	
gypsy-F	CAGATGGTTAGAGAGGattgatcgg	pHZM108
GUS-R	GTATAAAGACTTCGCGCTGATACCAG	
T7-F	TGCTAGTTATTGCTCAGCGG	pET28a
T7-R	TAATACGACTCACTATAGGG	
Primers for constructing vectors with enzyme digestion-ligation		
pGEX-F	GGGCTGGCAAGCCACGTTTGGTG	pGEX-6P1
pGEX-R	CCGGGAGCTGCATGTGTCAGAGG	
pMDC43-BnbZIP68-F	tgaactatacaaaggcgcgccaATGGGTAGTAGCGAGGTGGAC	pMDC43
pMDC43-BnbZIP68-R	ctctagaactagttaattaaTCAGGCTGAGTTGTTGTAGGAACCAC	
