# Supplementary material for: BnaC04.bZIP16 can be phosphorylated and inhibited by BnaA06.SnRK2 and negatively regulates the accumulation of fatty acids in Brassica napus
Source: Plant J. 2025 Oct 3;124(1):e70506. doi: 10.1111/tpj.70506 (PMC12494420; doi:10.1111/tpj.70506)
Supplement: Supplementary file 3 — Table S2. Sequence in paper. [file TPJ-124-0-s002.docx]

**Table S2 Sequences in article.**

**>BnaC04.bZIP16 CDS**

| ATGGCTAGCAATGAGATGGAGAAATCTAGTAAAGAGAAGGAACCTACAAC | 50 |
| --- | --- |
| GCCTCCTCCTCCTCCCTCTTCACAGGAGCCTTCCTCGGCTGTGAGTGCTG | 100 |
| GCATGGCTACTCCTGATTGGTCTGGCTTTCAGGCATATTCTCCTATGCCG | 150 |
| CCTCATGGTTTTGTGGCCTCAAGTCCACAACCTCACCCTTATATGTGGGG | 200 |
| GGTTCAGCATATGATTCCTCCTTATGGAACTCCACCTCATCCGTATGTTG | 250 |
| CAATGTATCCCCCAGGTGGCATGTACGGACATCCTTCAATGCCTCCGGGA | 300 |
| TCTTATCCATATAGCCCCTATGCTATGCCTTCTCCCAATGGAATGACCGA | 350 |
| AGCTTCTGGGAATACTACAGGTGGCACTGAGGGTGGTGATGCTAAACAAT | 400 |
| CCGAAGTGAAGGAAAAATTGCCTATCAAAAGATCAAGAGGAAGCTTGGGA | 450 |
| AGTTTGAACATGATTACAGGAAAGAACAATGAGCCTGGGAAAAACTCAGG | 500 |
| AGCATCAGCTAATGGAGCTTACTCTAAAAGTGGAGAGAGTGCTTCTGATG | 550 |
| GTTCAAGTGAAGGAAGTGATGCGAACTCTCAAAATGGCTCGGGATCTGGA | 600 |
| CAAGAAGGGAAGGATGAGAATGGTGATTCTGCTAATGCCCCCCGGAATGG | 650 |
| TACACCGGTGAGCCAGACCGTCCCAATCATGCCAATGCCAGCTGCAGGCC | 700 |
| CACCGACGAATTTAAACATTGGGATGGAATATTGGGGCGCTCCTACTTCA | 750 |
| GCAGCTATCCCTGGAATGCATGGGAAAGCATCTACACCAGTTCCTGGAGT | 800 |
| CGCTGCTCCAGTCTCTCGAGATGGTGGCCATTCACAACCCTGGTTACAGG | 850 |
| ACGATAGAGAAATTAAGCGACAGAAAAGGAAGCAGTCCAATAGGGAGTCT | 900 |
| GCTAGAAGATCCAGATTGCGTAAACAGGCGGAATGTGATGAACTGGCACA | 950 |
| ACGAGCTGAGTTGTTGAATGAAGAAAACGCAAGCCTGAGAGCAGAAATCA | 1000 |
| ACAGGCTCAAAAGCCAGTGCGATGAGCTCACCTCTGAGAATACCTCTCTC | 1050 |
| AAGGATCAGCTTTTATCATTCCCTTCACTTGAAGCCATTAACATGGACAA | 1100 |
| GGACGACCAAGAACCAGAAGCCTATCAAACAGGCGTCACAGAGAGAAAGG | 1150 |
| TTGATGATACCTACAAAAACTCAACGTGA | 1200 |

**>BnaC04.bZIP16 promoter**

| \| ttccgatacatagaaaagaactgtacctctaacttgtgcacatgatatag \| -2500 \| \| --- \| --- \| \| ctccaagaatcacgaaatttcatccttctctggaacatgatccagttggg \| -2450 \| \| cgtaaatccaattcctctcttcctcaatggcttcttcagatatctgaatt \| -2400 \| \| Tattaagaaaatcaatcgtccagatttcatcatatctctctgt  ac \| -2350 \| \| ttattaagatttggttccaattcatcttcaatccttagagtagacaccaa \| -2300 \| \| atcattttcccgtagtttttataataagcgttcagatcagttccttgcat \| -2250 \| \| gtatttccttttcttgacaccgttcctcactatcatcttcatcgataatg \| -2200 \| \| tagtctctcaattcatcattaacatcagatttctttaaacgtttggactt \| -2150 \| \| ggtcattttctggagattgttgttgttttcctagagaagctgataatcgt \| -2100 \| \| cttcgtgaagattgaaatcatctttcttttctccatcatgatgatcatca \| -2050 \| \| ctacattgcatcttttctccttcaacgtcatcaccttccattaaaaaccc \| -2000 \| \| atcaccatatatagtcgtaatgatcttcctctttaacaatctcctactct \| -1950 \| \| ctgtcgaaaacaaatcggaattttcagaaaagaaaagaagaacaataaac \| -1900 \| \| tcaaagggacttagattttatctacctttgtcatggtggacgttgttcct \| -1850 \| \| cgccatgactagttcaagaagaacaaaaagccctagagggttagggtttg \| -1800 \| \| gagcagcgctttttatggaacaattttttttattagttgcacactgtcat \| -1750 \| \| gcagtgatttttagtgtaaaaggccaattaaagcttctttttctattttt \| -1700 \| \| caaatcctttgggtgtttttaccacatgtgaaagtgaacaacaaatctga \| -1650 \| \| acgttgtaacatgcaacggtctatacataaagtacattctcgtctcgttc \| -1600 \| \| cgtaagccaagttctgtaaatgaagcttcgtgagattccttctttgtaat \| -1550 \| \| gttgattatgttctctgttcgatttggacaacaaaacaaattcaaaacta \| -1500 \| \| ttatatgaagaacaacaaatcgaaatccccaaatttcaattcgaaaacct \| -1450 \| \| taagaagagaaaattcccaaattttgaagaggaatcatactgaaatggcg \| -1400 \| \| tcgttggagaatcaaatcttcgtgcacttcgtcggagattatgacagtat \| -1350 \| \| tcttcgccggagatgaatggaacgaaatcgaagtcgacgttacgtggtct \| -1300 \| \| tcgccgccgtgagagagagcgaagagaagagattcaccgccgtgagagag \| -1250 \| \| atcgaagaggagaggtttctgagttttttttcggttgttttatttaatat \| -1200 \| \| aaaacaagggtattaaggatatttagtatactaataaatgttatttttgt \| -1150 \| \| gacttttctcttctagtgctatttttgagacaaaaattcaaaaatgctat \| -1100 \| \| tatggagattgcccaaaattttacataataaatttcaaaaattttagaaa \| -1050 \| \| gacagataagtccctatacatattgtatataattttatgttacatgaatg \| -1000 \| \| tttttaagaatttacgcattcgagctatcacgttcaaagttagagcggtt \| -950 \| \| acagtcgttcatacctttaataagaaatttcattgttttattatataact \| -900 \| \| aatcataattaaaaacaaatttcaataattttttatttatcgtaataaaa \| -850 \| \| aaatagtttatattgtgtttatctaaaatttacatcataaagttttaacc \| -800 \| \| tttttaaaaaagatgaacccttaatgaataatttgatttttgaatataat \| -750 \| \| taaccataagtaaaaacgaatttcagtgacttgataattataattataac \| -700 \| \| ccttaataaatagttcatagtattgttaataacaatttagggtatcaaat \| -650 \| \| aacaagttaaaaatttgaaggcaaaaaaaaaaaaaaatttcaacaaggta \| -600 \| \| aaacaaaaggactcagttttgaatctggactatgaatcaaaaataagttt \| -550 \| \| gaaggaaggggttttaatataaataaatacttaatataggcgaaagaagc \| -500 \| \| aactcgtattcctcctcgtgaattccacgccccctccccctttccttctt \| -450 \| \| cttctccgttggaaactcacaaacatatcaaccaattttgtctttggtca \| -400 \| \| aacacattgattccctcaatctcttgcaaatttcaaagctcctagattca \| -350 \| \| caccgcatacccagttttgtgctcagagttttcagacatcacccggtcgt \| -300 \| \| taggtatttctatcgcatctcaaagttcgtgtctttgggggtagattctg \| -250 \| \| cgttttttttgtctgaattcgacttgttttttctatcactacctacaacg \| -200 \| \| ccaatttgattgagaaagagacgacctttatttgttaattatcagtgtga \| -150 \| \| atgttgaactgctggatgtggtgggggtggtttatgcattaagtagatct \| -100 \| \| cgtggtgtattagctgagctcgtctttgcaggtacctctcactggtgata \| -50 \| |  |
| --- | --- | --- | --- | --- | --- | --- | --- | --- | --- | --- | --- | --- | --- | --- | --- | --- | --- | --- | --- | --- | --- | --- | --- | --- | --- | --- | --- | --- | --- | --- | --- | --- | --- | --- | --- | --- | --- | --- | --- | --- | --- | --- | --- | --- | --- | --- | --- | --- | --- | --- | --- | --- | --- | --- | --- | --- | --- | --- | --- | --- | --- | --- | --- | --- | --- | --- | --- | --- | --- | --- | --- | --- | --- | --- | --- | --- | --- | --- | --- | --- | --- | --- | --- | --- | --- | --- | --- | --- | --- | --- | --- | --- | --- | --- | --- | --- | --- | --- | --- | --- | --- |

**>BnaA06.SnRK2 CDS**

| ATGGACAAGTACGAAGTTGTCAAGGACTTGGGAGCTGGGAACTTCGGTGT | 50 |
| --- | --- |
| TGCTCGTCTCCTTAGGCATAAAGAGACTAAGGAGCTTGTTGCTATGAAGT | 100 |
| ACATCGAGAGAGGTCGCAAGATTGATGAGAATGTGGCGAGAGAGATTATT | 150 |
| AATCACAGATCACTTAGGCATCCTAATATCATCCGATTCAAGGAGGTGGT | 200 |
| TCTGACTCCAACTCATCTTGCTATTGTAATGGAGTATGCTTCTGGAGGAG | 250 |
| AGCTCTTTGAAAGAATCTGTAATGCTGGTAGATTCAGTGAAGCTGAGGCT | 300 |
| AGATACTTCTTTCAGCAGCTGATTTGTGGAGTGGATTACTGTCATTCCCT | 350 |
| GCAAATATGTCATAGAGATCTGAAGCTTGAGAACACTCTGCTCGATGGTA | 400 |
| GTCCTGCACCACTGTTGAAAATCTGTGACTTTGGTTACTCCAAGTCATCT | 450 |
| CTGCTTCACTCTAGACCTAAATCAACTGTTGGAACTCCAGCTTACATTGC | 500 |
| ACCTGAAGTTCTCTCTAGAAGAGAATATGATGGGAAGAATGCGGATGTTT | 550 |
| GGTCTTGTGGTGTGACTCTTTATGTGATGCTGGTTGGAGGTTATCCCTTT | 600 |
| GAAGACCCTGATGATCCCAGAAACTTCAGGAAAACAATCCAACGTATAAT | 650 |
| GGCTGTCCAGTACAAGATCCCGGACTACGTTCACATATCTCAGGAGTGCA | 700 |
| AACACCTTCTCTCTCGCATCTTTGTCACTAATCCAGCTAAGAGGATCACA | 750 |
| CTTAAAGAGATCAAGAAGCACCCGTGGTTCTTGAAGAACTTGCCTAAGGA | 800 |
| GCTTACGGATTCTGCTCAAGCAGCGTACTACAAGAGAGACAACCCAAGCT | 850 |
| TCTCTCTTCAAAGCGTTGAGGACATTATGAAGATCGTTGGAGAAGCAAGG | 900 |
| AATCCAGCTCCGTCTTCTAGTGCCGTCAAGGGCTTTGATGAAGATGATGA | 950 |
| AGACGAGGTTGAAGAAGAGGAAGAAGAAGAAGATGAAGAAGAAGAAGATG | 1000 |
| AAGAGGAAGAAGAGGATGAATATGAGAAGCATGTCAAAGAGGCACATAAG | 1050 |
| AGCCAAGAGTCTTCTAAAGCTTAA | 1100 |
|  |  |

**>BnabZIP16s gRNA1**ccattcacaaccctggttac

**>BnabZIP16s gRNA2**ttgcctatcaaaagatcaag

**>BnaA08.OLEOSIN1 CDS**

| ATGGCGGATACTGCTAGAACCCATCACGACATAACCAGCCGTGACCAGTA | 50 |
| --- | --- |
| CCCGATATTGGGCCGAGACCGAGACCAGTATCCTTACGGACGATCAGACT | 100 |
| ACCAGACGTCGGGCCAAGACTACTCCAAGACTAGGCAGATTGCTAAAGCT | 150 |
| GCTACCGCAGTCACCGCCGGAGGGTCCCTTCTTGTCCTCTCCAGTCTCAC | 200 |
| CCTTGTCGGAACAGTCATTGCTTTGACTGTTGCCACTCCTCTGCTTGTTA | 250 |
| TCTTTAGCCCAATCCTCGTGCCGGCTCTCATCACCGTAGCACTTCTCATC | 300 |
| ACTGGCTTTCTCTCCTCTGGTGGGTTTGGCATTGCAGCTATAACCGTCTT | 350 |
| CTCCTGGATCTATAAGTACGCAACGGGAGAGCACCCACAGGGGTCAGATA | 400 |
| AGTTGGACAGTGCAAGGATGAAGCTGGGAACCAAAGCTCAGGATATTAAA | 450 |
| GACAGAGCTCAATACTACGGACAGCAACATACAGGTGGTGAGCATGACCG | 500 |
| TGACCGTACCCGTGGAACCCATCACACCACCACTACTACA | 550 |
|  |  |

**>BnaA08.OLEOSIN1 promoter**

| ctcaaaaagtgaactatatgagaaatgcaataagttagatggtaatatgt | -1500 |
| --- | --- |
| gtaggcaaaaccagagacggtacagaaagtgagtgacattgtaaaggaac | -1450 |
| aattggctttgtccgcagacactgcactcactgccgagtcaaagttctct | -1400 |
| gctcttggcgctgattctctagacaccgtatgttattatactctctcact | -1350 |
| ctctctctcctttcatatcaacgattattttgttagttatttagttattc | -1300 |
| aaataatattgtcatgtgaatatatggttacaggtggagatagtgatggc | -1250 |
| tttggaggaaaagtttgatataagtgtggaggaaactgatgctcagaaca | -1200 |
| ttacgacgattcaagaagcagctgatttgatagaggatcttgttcagaag | -1150 |
| aaacctgcggcttaggcttcctagactcttttttttttttaatttccttc | -1100 |
| tgtcctttcttggtttcagagcatttatttttgctatattagaccgttgg | -1050 |
| ttacatttctttttctggaagttacgattggatgagacaacttactgaag | -1000 |
| ctgttccattgtagagttggaaaaggtcactattattatttttttttttt | -950 |
| tgaatgaatgttaaattttattcaatccaaaaaacccctttgtttacata | -900 |
| tttacaatgctacatatataagaaatttttcactctttttttaatctata | -850 |
| ctcatctatgaacttgcatctaatcttgttgaaaaccagaaactgagccc | -800 |
| attctccatctttgcgtctcccatttgctgaactgaagcaaatctgtttc | -750 |
| tattataatattattattattcttatgctgattattcttcaggtcatgtt | -700 |
| ctatcaaaggtgtatcagctcactggtgtaggttagcaacatgggtgtat | -650 |
| aacagtataagtggtgtacatataaagcttatgttagcgagattgtatta | -600 |
| gagtaacatactgtacagttttaattaatgagaagtttcaacattcataa | -550 |
| caaattttcttccatctcatcttcttcaagctctcgttgctgtgaatctt | -500 |
| gatatggtatcttgatatagttcttccttgttattgtaatcaatgtaata | -450 |
| tgtaactttatggtatgctcaaaaatagtcttgggtttacgttcgatatt | -400 |
| tgactttttgttgttaagaagaaattcatggaaaggccttgaaagccctg | -350 |
| aaaccaccctctgataatcagcccaatggtccgttttttaagtttacgtt | -300 |
| cggttcattttgccaacgccacattcacaggaacgagacaaaaacacaaa | -250 |
| cgtgtcctcaagtagacccctctagtttacacctgcagcgtctgcatggt | -200 |
| gacgccaccaacacgtggatactattgcatgatgacgccattgacacatg | -150 |
| actctctttccccctcccttctcttcatatatctaatcaatttacaacaa | -100 |
| tcatagctacttcaaaacataaactcatctctctcttcaacaagatcata | -50 |
|  |  |

**>BnaC03.PDH_E1 BETA CDS**

| ATGGCTGCGAGAATCCATGGAGGAGCTGGAGCTGGAGCTGCTACGGCCTT | 50 |
| --- | --- |
| GTCGACCTTTAATCCCAAGAAACTCGTTGCTCCTTCCCGCACCAATCTCC | 100 |
| CAGCGAGGAGCAGCAAGAGATGCATTGTGGCTGCTGCTGGATCTGATGCG | 150 |
| AGTAAGAGCCTCAGAGTTGGTCACTCTCAGAAATTGATTGCGAATGCTGC | 200 |
| TGTTGCGACGAAGGCGGAGACATCTGCCACCACTGGCACTGGTCATGAAC | 250 |
| TACTGCTTTTCGAGGCTCTTCAGGAAGGTCTGGAAGAAGAGATGGACAGA | 300 |
| GATCCACATGTATGTGTTATGGGTGAAGACGTTGGCCATTACGGTGGCTC | 350 |
| CTACAAAGTAACCAAAGGCCTTGCTGATAAATTTGGCGACCTCAGGGTTC | 400 |
| TCGACACTCCTATTTGTGAAAATGCATTCACCGGTATGGGAATTGGAGCT | 450 |
| GCCATGACTGGTCTAAGACCTGTCATCGAAGGTATGAACATGGGTTTCCT | 500 |
| CCTCCTGGCCTTCAACCAAATCTCCAACAACTGTGGAATGCTTCACTACA | 550 |
| CATCTGGTGGCCAGTTCACAATCCCGGTTGTCATCCGTGGTCCCGGTGGA | 600 |
| GTGGGACGCCAGCTCGGCGCTGAGCATTCGCAGCGGCTAGAATCTTACTT | 650 |
| CCAGTCCATTCCTGGGATCCAGATGGTTGCTTGCTCGACTCCTTACAACG | 700 |
| CCAAAGGGCTGATGAAAGCCGCGATCAGAAGCGAGAACCCTGTGATTCTG | 750 |
| TTTGAGCACGTTCTGCTTTACAATCTCAAGGAGAAGATACCGGACGAAGA | 800 |
| GTACATTTGTAATCTTGAAGAAGCTGAGATGGTCAGACCTGGAGAGCACA | 850 |
| TCACCATCCTCACTTACTCGCGGATGAGGTACCATGTGATGCAGGCAGCT | 900 |
| AAAACTCTGGTGAACAAAGGGTATGACCCTGAGGTTATCGACATCAGGTC | 950 |
| GCTGAAACCGTTTGATCTTTACACGATTGGGAACTCGGTTAAGAAAACGC | 1000 |
| ACAGGGACGTTCCTACACCGTACGCTGGTACACTGGAGGAGTGGACCGTT | 1050 |
| GTTCAACCGGCTCAGATCGTGACCGCCGTTGAGCAGCTTTGCCAG | 1100 |
|  |  |

**>BnaC03.PDH-E1 BETA promoter**

| ggtatatcgcggaaagttttgggctgcattttcattattatttaatagtc | -1500 |
| --- | --- |
| taagcttggagtgggcttggtgggtacccacatcagtcctggattcgagg | -1450 |
| aactaaattatcactacttggccagtctgggtttgggcttcggcccaagt | -1400 |
| ggtttacatggtgggccataacagatgattggtccacccctggcattagt | -1350 |
| cggaaggtattccaaactcgggtcaggcagtgtggtacactttggggtta | -1300 |
| gtccactctgtagcactaagtgcgttcctcccggagccgaccggatcagc | -1250 |
| cgatagggtttataaaaaaaaaaaaaagcatataattgttgggtaatata | -1200 |
| aattcatacctattaaaaccaaccaaaaagtaaggttcggaataaataaa | -1150 |
| taaggtctagccgacgtaaagttcgaatgaagaaaacgacatctagctat | -1100 |
| cttaaagaaatagagtcaagaaatgaaaaggttcaaccagcataaggtcc | -1050 |
| aaaaagaaaaaaatgtccaactagcttagcataagccgtaaggtccgaaa | -1000 |
| agaaaaatattcaatcagtttaaggcctcaaattcaaaaacaaaatcgac | -950 |
| ttaaggtagtgctaggtttccatgctagcagaggatgcttctttcactgt | -900 |
| tacacacgtccatcctatgcggacactagacgggatcaccttaccccacg | -850 |
| gtaacagccagagccggacctaacgttacgggccctatacaaaattgaaa | -800 |
| ttttatctaatttttagtaaaaaaaatattttatattttgtaaaagatta | -750 |
| tttttaaaataaataatcataaattttatctcataatctaactttactta | -700 |
| atatctttaacatttttatgttttatatagtatatatttatatgtatata | -650 |
| gttttttttttaaattgggggccccatgaagtggaggccccattccaatg | -600 |
| tttcatctcagtaggttcaagtccggctctggtaacagctcaacgcaaca | -550 |
| tcatggaagtataatgggaaaaacatgtgcaaaaagaacacctatccaga | -500 |
| agaatttcccacttggtcaagcagtaagacattgacatatcattgggccg | -450 |
| gtacatttgaggcccatccccgcatccaaaaccaatgaaaggtcataagt | -400 |
| attgttaaatgtaattgggttggtccggggtggttaagcgaacccgtcct | -350 |
| aactcctctacaagcttgttcatggagttatatatactccacaatttaga | -300 |
| tacaaacacgtgtctactataatcgtaaatgaacacgtggcatacttata | -250 |
| ctggtttcatacaaaatagggggccacaggagattagaagtggtgcatgg | -200 |
| cgaagaaaacaatgtacaacaaacatgtggattggatcgaacaatagaca | -150 |
| cacaaatttttatttatttttacagaagaaatcaatttcacagctattct | -100 |
| cactagtccttcgatttctccgcttcgtcactctccctggggaaaaaaaa | -50 |

**>BnaA08g14540D_BnaA08.OLEOSIN1(up_Stream_Len 1500 Down_Stream_Len** 300)ctcaaaaagtgaactatatgagaaatgcaataagttagatggtaatatgtgtaggcaaaaccagagacggtacagaaagtgagtgacattgtaaaggaacaattggctttgtccgcagacactgcactcactgccgagtcaaagttctctgctcttggcgctgattctctagacaccgtaatgttattatactctctcactctctctctcctttcatatcaacgattattttgttagttatttagttattcaaataatattgtcatgtgaatatatggttacaggtggagatagtgatggctttggaggaaaagtttgatataagtgtggaggaaactgatgctcagaacattacgacgattcaagaagcagctgatttgatagaggatcttgttcagaagaaacctgcggcttaggcttcctagactcttttttttttttaatttccttctgtcctttcttggtttcagagcatttatttttgctatattagaccgttggttacatttctttttctggaagttacgattggatgagacaacttactgaagctgttccattgtagagttggaaaaggtcactattattatttttttttttttgaatgaatgttaaattttattcaatccaaaaaacccctttgtttacatatttacaatgctacatatataagaaatttttcactctttttttaatctatactcatctatgaacttgcatctaatcttgttgaaaaccagaaactgagcccattctccatctttgcgtctcccatttgctgaactgaagcaaatctgtttctattataatattattattattcttatgctgattattcttcaggtcatgttctatcaaaggtgtatcagctcactggtgtaggttagcaacatgggtgtataacagtataagtggtgtacatataaagcttatgttagcgagattgtattagagtaacatactgtacagttttaattaatgagaagtttcaacattcataacaaattttcttccatctcatcttcttcaagctctcgttgctgtgaatcttgatatggtatcttgatatagttcttccttgttattgtaatcaatgtaatatgtaactttatggtatgctcaaaaatagtcttgggtttacgttcgatatttgactttttgttgttaagaagaaattcatggaaaggccttgaaagccctgaaaccaccctctgataatcagcccaatggtccgttttttaagtttacgttcggttcattttgccaacgccacattcacaggaacgagacaaaaacacaaacgtgtcctcaagtagacccctctagtttacacctgcagcgtctgcatggtgacgccaccaacacgtggatactattgcatgatgacgccattgacacatgactctctttccccctcccttctcttcatatatctaatcaatttacaacaatcatagctacttcaaaacataaactcatctctctcttcaacaagatcataATGGCGGATACTGCTAGAACCCATCACGACATAACCAGCCGTGACCAGTACCCGATATTGGGCCGAGACCGAGACCAGTATCCTTACGGACGATCAGACTACCAGACGTCGGGCCAAGACTACTCCAAGACTAGGCAGATTGCTAAAGCTGCTACCGCAGTCACCGCCGGAGGGTCCCTTCTTGTCCTCTCCAGTCTCACCCTTGTCGGAACAGTCATTGCTTTGACTGTTGCCACTCCTCTGCTTGTTATCTTTAGCCCAATCCTCGTGCCGGCTCTCATCACCGTAGCACTTCTCATCACTGGCTTTCTCTCCTCTGGTGGGTTTGGCATTGCAGCTATAACCGTCTTCTCCTGGATCTATAAGTACGCAACGGGAGAGCACCCACAGGGGTCAGATAAGTTGGACAGTGCAAGGATGAAGCTGGGAACCAAAGCTCAGGATATTAAAGACAGAGCTCAATACTACGGACAGCAACATACAGGTGGTGAGCATGACCGTGACCGTACCCGTGGAACCCATCACACCACCACTACTACATAAatcacgcaacagcccccagtaatgtcgatgtcaggggagtctagttcatgaggaataaggtgtttagaatttgatcagggtgggtaataaaaggggaagcatatatcatcagtttttttttctttttttgttataaataatgtgcaagtgtgtttctcttgtcaaatggtaccatgttttgttgtgttattctctttgtggtaaattgtaaaccttttgctgtactttctttgccttgtttgtaagttgtaacgtctatatggagaagtcttgttcgtccaacttatgtttggtctgcct

**>BnaA08g09710D_BnaA08.SPH2 (up_Stream_Len 1500 Down_Stream_Len 300)**

attatagtaattaaagtcatactaatttgagtataaattagtctttccagtggaattagtttaattggagactggttttattatgcaaaaaaattattacaaaattctaaaagattattaatttatgttatgaaatttttaaaagattattagtttttccagtggcattagtttgattacagactggttttattttttatttattcaataaaaaaaaaatttaatttcgcattttaaatatggtgttaaacacatgtatgtaatattaatctgctggagatgaataccaatcatgcctttaaaaaaatttaccgctaagatcttaccgccaaaattctagaataataaaatttaaagctacatcaaaaattttaaaactacagtatttaccaatcagacccacaaatctgagaaaacaagaggaatttcaatgaagagaagtgatttagtacaaagtttatctagttgttctaaaaacatcaggagcttgattttagagttcattggatcccaaaactaaagatttttatgtcatgttaaagatttaagtatggaaatcacattagaatgtgtatgggtgcaacatctttttttggttggatcatacaaataaaggattgttttttattctttttaaacaagagctcaatggtgttaagataataaaatatgttaggcattttcttcctcttcagtgacattatggtgacgataactgcttgcatttgcaactccaaattgttatgttcatcacaagtattatcttgggatgtcacgtacgtagaaattccagagaaatgcacaattttcatcagctatatgactacaaagagaataatagaaagatttcatttaaaattatttcacaatgtatgtagccaattgagattataagcaaaatgtaccttttaactcaaatcgtctattaccggaggatatagcagccattggaagataggagtggaaagaaaaagcattgaacaaatatgcattaagttaggatctcatcaagagctagtgaatttactcaacaacataatggaatctacctgaagcagcttgatatccagagacccagtttcctgtttgaaaatcatacacgtgaatgatgttggtcaacatagtctacatatatatatatatatgatattcttctagctacaccttctggcttaattatcttagattttttacagctccaagaggagggtgtacatctagaaagaagacttgatagatgactaatctcttgaatcagtaaaacggtgacgttttggtggttacgactttgtacaagtcgtcctctttgtacacttgtatcgtaactaaagatcgttacgccacgtcatctcatccattcgcttaatacagcgcgtgactgtcacctcccatctgatcagacaaaagtagtcacaaagtcaattttgttgttcttaactcacctgcgaagctaacaaagaatctctctctctctctttctcacggagaactgtttgtgttATGGCTCCGGCGGAGGTAATAGTCGTCGACCTAGTCTTAATAGACGGCGAACTCGGGATGGTGAAGTTGACCGCCGATGGAGTTTTAGAAGCGATCGAGTACGGTGAACCCAGCCGTTACTGGACGGTGAAGAAAGACGTTCTTGGTTTCGTCGTGGAAGGTAAATATATTAGAATAAAAACAGTGGTGGAGAGAGAGGAAGGTATCTGTTGCGGAGAGTTTGGTGGAGATTACTCCAGAAAAGACTTCGTCTTTGAGCCTTTCTCTGAAGATGCTAAGAATAGATTCTGCTTCAAGCTCCGTCACTACCTTGACTCTCTCGGTCGGCCTAAGAGGTTGCTTGTGTTTGTGAACCCTTTTGGCGGGAAGAAATCTGCTATAAAGATATTTGAAAAGGAAGTGAAGCCATTGTTTGAAGATGCTGACATTCAACTTGATGTTCAAGAAACAAAGTATCAGTTGCATGCAAAGGAAATGGTTAGGTCCATGGATGTATCAAAGTATGATGGTATTGTTTGTGTCAGTGGCGACGGTGTCCTTGTTGAGGTTGTAAATGGACTGCTTCAAAGATCAGACTGGCAAACTGTCTTCAAATTGCCAATTGGAGTGATCCCTGCAGGAACTGGTAATGGCATGATAAAGTCATTGTTGGATGCGGTTGGGCTTCAGTGCTGTGCAAATAGTGCTACTATCTCTATTATCCGAGGGCATACACGTTCTTTAGATGTGGCAACTATCTCACAAGGAAATACCAAATTCTTCAGCGTCTTGATGCTTGCTTGGGGTTTAGTGGCTGATATAGACATAGAGTCAGAGAAGTTCAGATGGATGGGTAGTGCTCGTATGGACTTCTATGCCGTTCAAAGGATAATAAGTTTAAGACAATACAATGGACGAGTTTTGTTTCTACCGGCTCCTGGGTTTGAAAGCTATGGGCAGCCAACCAGTTACCGTCTATACAAAGAGCCACCTGTTAAGGCGCTTGGATACCAAGGACCTGATACTAAATTTGAAGATGTTGAATGGAGAGAAATCAAAGGCCCTTTTGTTTCAGTATGGCTTCATAATGTTCCCTGGGGTGCTGAGAACAATTTGGTTGCTCCTGCAGCAAAGTTTTCTGATGGCTTCCTGGATTTGATCGTCGTGAAAAACTGTCCTAAGCTAGCTTTGCTATCACTTATGACACAGATAAGTGAGGGAACACATGTTCAATCACCATATGTTGCATATCTAAAGGTGAAGGCATTTGCACTTGAACCGGGGGCACTCGTAGACGAACCAGACAAGGAAGGAATCATAGATGCAGATGGAGAGGTGTTGGCAAGAGGAAGACGAACGTATAAATGCGAGCAAATTGCTTTGATGTCTTACGACAAGCTTCAAATTACAGTTGATCAAGGTTTAGCTACTCTCTTCTCTCCTGAGTACTGAtaaatattacaacactacacgtgagatgaagacacattcacacatcatatttcataaaaactgattgacgttttaattgtataataatctcttattgtaaatggtgttcccgtgtggttgtaatttaattgtttcacatgtacagatacattatttggaaatgagaatcacacttgttttgtgtgtttttttttctcattattaatcgtaaagattccattttaaaaaaaaattctgagagagattctgatcctttttttttggcttcaatcaaccaagcttatcaaaggcagttaaata

**>BnaC02g38800D_BnaC02.PKT1_KAT5 (up_Stream_Len 1500 Down_Stream_Len 300)**

ctgactgttagcaccgcccacttcccttgcaacttgtctccacacctcgttggctacttctcattcacacggcaaaaatgtagtaagccattgggctttatgtttatggatttatctcggcccatttagctttgctcctatttatattctccattcccccagttttagaattcggacatttgtaaataacaatgaatctaaaccaaggtccaagtgcgacaaaaccttgaagttacacttgtagaaatcttgtacacatttgatgaaacttttctttgtaatagatataacaaaaaaaagttaagtaacaataccgttagaagcaagaaagaaagggaagggtaggtagcatgagacgatgaaaagacatttaagaggaggttaggtaagaagagtggtggaggagcaaaagagtatgtgagtagtgttaaactccaaagccacgtcacttctttctctgtctctcttttgtttgttggttaaatttaattttgaacatttggtgtggtagctgctagcctactagctagctaccatcttctcttctctcaacatttgcatatatgttcaacattctctacatatatacctaccttttgtgactcttaattacagtagaagggtcttttactaagctcactcatcacatccggtaattttctcaagctctctcttattattttattttttcttgctaagaatgtagatatcatttaaagaaatgaactgcttaggtattacaaaatgtgattacaaattagaaaacctctttctctatatattttttttttttgccgtcacctctttctctattatgttattacaattcttgaatcatgcaatctttgcacactatcatgggaagctacgtgactaaactccgttttgtttgtatcccaaaactgatgcatgttatgagatggcatattctatttgtaaagctatttcttggagtcagtccattttatattctttatataagttttttcttcctaaacaaaccatattcaagcaatctactaaaaacatgacgttgttgccttctttctatccaaaacggcctgtagtttttcttttcccacgtgttcgtcaatgaatctactaaaagcatgacgttgttgccttctttcaactcaaaacgacgtgtagttcttttttttcctacgtggctggtttggcgctcattgccgtgtctaatcccgtctcaccagtaacggcgagaggttctgtgggggagaaaaacatgaaaaaagagacggacctaactgtaacgccgttaacttccttttttttaccaaacgacaagacaaggttggcaaaataacaaccagccaatcagacacagagtgggcagaaacgatcaatcgagaatattgaggatcctcaagccttcatatacttacaatgagtcttcctcatttcaaacagaacaaacccagttgaattttcccagaaagttttctcctttttttcttgaattaagcttaacagtgagaaaagATGGAGAAAGCCACTGAAAGGCAGAAGATTCTGATTCGTCATCTCAATCCACTTTCTTCTTCTCCTTCTCTTCCTAGCATTAATGATAATAAACCTACTCTTCTCTCCGCTGTGAACTGTGCTGCTGAGCTTTCCCCAATGGCTGCCTTCGGAGACGACGTTGTGATCGTTGCGGCGTATCGCACTGCCATCTGCAAAGCTAAACGTGGAGGGTTCAAAGATACTCTCCCAGATGATCTTCTTGCTTCTGTTCTCAAGGCTGTGGTGGAAAGGACGTCTTTGGATCCAAGTGAAGTTGGGGATATTGTTGTTGGTACCGTTATAGCTCCTGGCTCTCAGAGAGCCATGGAGTGTAGAGTAGCTGCTTACTTTGCTGGCTTTCCTGACTCTGTGCCAATCAGAACTGTCAACAGACAATGCTCATCAGGACTACAAGCTGTTGCTGATGTTGCTGCTTCCATCAGAGCTGGATATTACGACATTGGTATTGGTGCTGGAGTTGAGTCCATGTCGATTGATCACACTGCTGGAGGTGGCTTTCACACAACAAACCCGAGGGCACAAGAGTTTCGTGGAGCTCGTGACTGCTTGCTTCCGATGGGCATAACTTCTGAAAACGTAGCAGAAAGATACTGTGTCACAAGAGAAGATCAAGACATGGCTGCGGTGGAGTCTCACAAGCGTGCAGCAGCTGCAAACGCGTCTGGTAAACTAAAGGATGAGATAGTTCCTGTTGCGACTAAGATTGTTGACCCTGATACAAAAGCAGAGAAGCCAATTGTTGTTTCTGTTGATGATGGTGTACGTCCAAACTCAAACATGGCTGATCTTGCAAAGCTGAAGACAGTCTTTAAACCTAATGGTTCAACCACAGCAGTAGGCAATGCTAGTCAGATTAGTGATGGTGCTGGAGCTGTACTGCTGATGAAGAGGAGCTTGGCGATGAAGAAGGGACTTCCCATTCTTGGAATATTCAGGAGCTTTGCTGTTACTGGTGTGGATCCAGCTGTAATGGGTATCGGTCCAGCTTACGCCATTCCCGCTGCAGCCAACCTTGCAGGACTCAAAGTTAGCGATATCGATCTATTTGAGATCAATGAGGCATTTGCATCTCAGTATGTGTACTGTTGCAAGAAGCTAGAGCTGGATGTGGAAAAGGTCAATGTTAATGGAGGGGCCATTGCTATTGGCCATCCTCTTGGTGCTACAGGAGCTCGATGTGTTGCGACATTGTTGCATGAGATGAAACGGAGAGGGAAAGACTGCCGCTTTGGAGTTATTTCAATGTGCATAGGCACTGGTATGGGAGCCGCAGCTGTGTTTGAGAGAGGAGACTCTGTTGATGACTTGTCCAATGCGCGTGTGGAGACTAATGGGAGCGGTCACTAGaacaaggttgaataaggacaagcaatgaaagcactaagtcaaataaaaaagtgtgaagcttaaacagctctttcacattgttgaacaatgaacaatttgtcattctgagtttaaaatgtatcaactagtaacttgtgagtgaacaaagagtcaaatctaaaaagcaaagttacatctttcaccacacaagtctttaacatactaatcaatcctctcactctacatcttctggagctccaccaggcaccaactgcaacacaacaattttcaatacaggtttcttagcatttgacgattcaa

**>BnaC09g22540D_BnaC09.CLS (up_Stream_Len 1500 Down_Stream_Len 300)**

tatgttatttcgtttgttactattctatttaataatacttcatgttatatactatttacatattttgatatttggattaaagtttatatttactctttagggtttagtaattaatattttagatttagtttatggaatattttagttggtgccagggtgagcattacctcttttcttgttatttgttaattttagatatacatagtatagtatatttaaaccatttaataattttattaatatgaactataccatttaccatattctattctatttatgttttcatatgatgaaagagttatgtgtacatgcctataattttattatcatgttgtttactatctataagatacatatgaacattgaaaacaactgtagatgtcaagcatgtaaacacattatacttttctaatattatgctattcgataatttcattgtgtgtactattctatttagtttatttcataccattcagtttgttccatatcattcagtgttctatatcattatgcataaaagaccatcatttcgtctccatctacgacaattttttttttctttagccacgacgacgaccaccacaacacagccacaatatctattgatacaaaaagccattgtttcttccatatttatgactgtcacaccatcatcgtcaatgccataaccaccatcactgtaacaacgttttttttttttttttattttaagtgatatccttatgtgttatcaccgtcaaccgtcttagaaaaatgagaaaaccggaagaaaaaaagaaaaaaatgataggagatctaattatgtcaacatcattgccatattcttaatttttctttcaaatttgtctattacacaaattaacccttaaataattgtctttgcttttagcatcatttaatattttggatatatttttagtactatacatacatgttattaatatataatatttatctattatttaatttgaattaaaataaattatattgatttcattccattcttttaattatatttgtgttgttaaaaaagtatttgcaatgtatagagtgagccgtcctacttgattttacaaaaaaatatcagctaaataactgaaaacatgtataagcataaatatagagaaataaaaagaataaataaaataattcaatttattagaatagggaattttatgcttatacttaatttttgatttcattattctatgcatcattttattttgaacacctatacatcttcgtttcatttccatccagttacactctgaattttcaatccggttaatgtaccggttcgatataatatggcttggttaatgttccggttcagaataaaattattggtttggcatggtacataaagcttccacgtgtttatgaaacatcatacacgtggcccaagtcaaaaccctcaagcagcgaaaacctagtcgtcccgtcaccgtaaccaattcgaattccaatttaacgagaaataaaataagcgttagatgatcgATGGCGTTCTACAGATCTCTAAGAAAGCTTGCTGAAACCAATCCCCTTAAAAAGTCCAGACCATTCTTCACCGCCGCCGCAGCTTCCGGCGGAACCGTTTCTCCGATCGCACCGTCGTTTTCGCCGCTGATCCCACAGTTCACGAACCGGTTGTCTCCGCTTTCGAAATGGTTCGTACCTCTCCACGGACCTCTGTTCCTGTCTCCTCCTCCTTGGAAACTGCTCCAGTCCGCGACGCCTCTTCACTGGCGAGGAAACGGCGCCGTTTTGAGGGAGGTGGAGGCTCTGAGTGTTAGATTGGATCGGATTAGGAGAAGAACGAGGTTAGGGTTACAGTCTGTGACACAACACGTGGATGATAATTATTTGGCTAAGGAGGAAGATGGAAGCAGAGGAGGAGGGATAATGGAGAGTTTTGTGAATGTGCCGAATATGATATCTATGGCACGATTAGCATCTGGTCCTGTGCTTTGGTGGATGATATTGAATGAGATGTATACTCCTGCTTTCGTAGGGTTGGCTGTTTCTGGAGCTAGTGATTGGTTAGATGGTTACATGGCTAGGAGAATGAAGATTGATTCTGTGGTTGGCTCTTACCTTGATCCTCTTGCTGACAAGGTTCTTATTGGATGTGTTGCATTAGCAATGGTGCAGAAGGATCTTCTTCATCCTGGGCTGGTTGGAATCGTGGTGTTCCGTGATGTTGCACTGGTCGGCGGAGCGGTATATCTAAGGGCATTAAACTTAGACTGGAAGTGGAAAAAATGGAGTGATTTCTTCAACCTCGATGGTGCAAGCCCCCAGAAAGTAAAACCATTATTCATAAGCAAGGTGAATACAGTTTTCCAATTGGCTCTAGTCGCTGGTGCACTCCTTCAACCTGAATTTGGGAATCCAGATACACAGACATGGATCAATTATCTAAGCTGGTTAGTTGCTTCGACGACTATGGCTTCAACAGCAGCTTATGGAGCGCAGTACATGAAGAACAAACCTATCTCCATGATTAAGAGATCATAGtaacattgacttacttaacacactggccgcacaccggaaaggcctgaacgactcttactgtttccaggaatctgcagctacttttcaatttaaaaaaaaggaatgtgcggttaacaaatgaagcctgtgaatgagaaacgatgtctttgtctttttgttgtaaaaagagttgggctattggtttcttttctttttgttcttcctatagcttctggtaatgagagatgtctgaaagtggatgtttaaccttggagagaaataaatcgaatcctggcacatgcatctcttttatcagacaac

**>BnaC02g01400D_BnaC02.DGK1 (up_Stream_Len 1500 Down_Stream_Len 300)**

ttcaaaacacgctaatcattcaatatatcatatgtgttacttgtacgagtttctaaattataccagcctgatgtttcaattttcgtagcaattttcaaaattctgttgcagaaactgttgacacctgctacaattaaaatacacataattacgtatcaaacgataggtgacacgccagtctaaaacaaaataacattattatttttgcagtatgttaaagcaatcaacatatacgtcataacactacaaacacaaaaaaatattattcatataagaacactacgaaaacaagacacgattattattcatataagaacactacgaaaacaagacacgatatgaaaataacatttcgttttttgtagttattttaaataaatcaatataaatgtcacaagactcgcaacactactaactataaacatttattcgtattaattagaacactatgggaaagtacaagatataaaacgaatccaacttatccttttgttttggagtgggtcaatatcattcattcatacatgctgaattaattccaaatcgtgtcacgacagtaatgggcctgggcccattgccgacaaaaatttagtagggacacgtgggtattcagaatccgagattcatttatcctacgtggccaggccgtgtattacggatactgatagattataaccctctttctcgctcagaatctttccctttcataaatacttagacccagcgaattaaaaatatatataaactcgagcgcatctgctttcgaaaaagaaaaaaaaaaaaatttggattttggaattttccggtggagtgaaaagcttcgtgcttgggtgcgtcgattctagggtttgtttatgttctctcctttcgcttgatcgatctctatcgttatcatctgatcatcttcttctccgtcgcgtttggatgttagggtttcctgctattttatccccttttgtgattttcattgatttttggcgattgttgtgatcccagtttgttttttagttcgagtcgccgtattgagtatgcaggatcatgagcatttgagcagattgatgtattaagcacgaacaattcgcaggaaggtggattcgaagtatgattatttaattgcatctgtttgatttgagactgaatctgagtcaagtacgatcaataaagtttgaaactttgctgatttttaatgaccaaacatttactatatttcatttcattttgtttggtattatttgttttttgtttttttttgttttatagtgtttctttcattctcttatgaaagtgatttgttttcaagtttcgctgagtgacacgtgttttttcttgacagggctgaagagtttgttttctatagttgaagtgtgattgtactctggatgatgaggatatggactctgtatagtatattgatctcctctgtgtggacctgaccaagacaagaaatctttctgattggctccaactttaggcacgtgtctcgagttgcaacgtagatagATGGAGGACGATGGAGAAATGGGGATGTTCTCCAAGAATCCAGTTGAAATGGTGGAGTCTCGCGGTGTAATGTTCTCTTGCTTTGTTGCTGCTCTTGTTGGTATTTTGACCATAGCCTACACTGCTTTCCAGTGGAGAAGGAATATCAACTTGAGTTGGACAAAAGCCATAGCCAGGTCGAAGAAGAATCCCAAGGCGCGGCATAAGACTCCCGTTGCTCCGCACAGCTGGGAGCTTCACTCTATATCTCGCGCCAAGAACTTGAACTGCTGTGTTTGCTTGAAGTCGATGTCGCCGTCGCAGACGATTGTAGCTTCTGAGACTTTTGTCCATAGGTGTACTATATGTGGAGCAGCAGCCCATTTTAGCTGTTCGTCAAGTGCTCCTAAAGATTGCAAATGCGTCTCCATGGTTGGGTATGAGCATGTGGTGCACCAGTGGGCGGTGCGGTGGACAGAAGGTGCTGATCAGACTGATGATTCTTCGTTTTGTAGCTACTGTGACGAGTCGTGTAGTAGCTCCTTTCTTGGAGGTTCTCCTATATGGTGCTGCTTGTGGTGTCAGCGTCTTGTCCATGTCGACTGTCACAGTAATATGTCGAGTGAAACAGGGGACGTTTGTGATCTAGGCCCTCTTAGAAGGTTAATTTTGTGCCCTCTTTATGTTAAGGAGTTGACTCGGAATCCATCTGGAGGATTTTTGAGCACGATCACGCATGGTGCAAATGAACTTGCATCTACTTACGAGCTAGCTGACTTGCCTTCAGATGCAAGACCGTTGTTGGTTTTCATTAACAAAAAGAGTGGTGCTCAACGAGGTGATTCCCTTCGTCAGCGCCTTAATCTTCTTCTTAACCCCGTGCAGGTCTGTGAATTGAGTTCAGCGCAGGGACCAGAAGTGGGACTTTTCCTCTTCAGGAAGGTTCCTCACTTTAGAGTTCTTGTTTGTGGTGGAGATGGCACTGCTGGTTGGGTATTAGATGCCATAGACAAACAGAATTTTGTATCTCCTCCTGCGGTTGCTATCCTGCCTGCTGGAACCGGGAATGATCTTGCCCGGGTATTGAACTGGGGTGGTGGTTTGGGTTCTGTTGAGAGACAAGGAGGCTTATCTACAGTATTACAGAACATAGAGCATGCTGCAGTCACCGTCCTTGATCGTTGGAAAGTATCGATTCTAAATCAGCAAGGAAAGCAACTCCAGCCGCCAAAATATATGAACAATTATATAGGTGTTGGGTGTGATGCGAAGGTAGCCCTTGATATTCACAATCTACGGGAGGAGAATCCAGAGAGATTTTATAGCCAGTTTATGAACAAAGTCCTATATGCTAGAGAAGGTGCAAGGAGTATAATGGACAGAACGTTCGAAGATTTCCCATGGCAAGTTCGTGTTGAGGTGGATGGTGTTGAAATCGAGGTTCCTGAGGATGCTGAAGGAGTGCTTGTTGCAAACATCGGAAGTTACATGGGAGGTGTGGATTTGTGGCAGAATGAAGATGAAACATATGAAAACTTCGATCCTCAATCCATGCACGACAAGATAGTAGAAGTTGTGAGTATATCTGGAACATGGCACCTTGGCAAACTTCAGGTTGGGTTATCTCGTGCCAGAAGGTTAGCTCAAGGACAATCAGTCAAGATACAACTCTGTGCGCCGTTGCCTGTGCAAATCGATGGAGAACCTTGGTTTCAACAACCTTGTACCTTAACCATATCGCATCATGGCCAGGCTTTCATGCTGAAGAGAGCTGCAGAGGAGCCACTGGAAAATGCAGAAACCAATGAAGTGATCAACGCTTCACAGAAACGAGCTCTGCTTCAAGAAATGGCTCTACGGCTAACTTGAaaaagaaaccttccctcttcaacaaccaatcaaccatcattggctagtcactgtctaatagaagttttaaagtgtgtgtttacatgtcaaatccgttgaatgaaaaagcgggttacgtgtggaagtaggaaaaaagaaaatggaacccggttccaagaattttggctgttctcaacgtgttgtgtcataaggtcatgtgtggaactgatacaaagcttgttaaattatctatcatcttttttttatattaatattacatctccagaatgtcttatctcttctgtattagcgttaatcacg

**>BnaC05g41530D_BnaC05.DGD1 (up_Stream_Len 1500 Down_Stream_Len 300)**

tccaacttacgtgggtcattcctctgggtccggttaggcagcggtccacttcacccgggagggttttacctgggctggggacaaggtccaacacccagtaccgcatttggtgacagaatgggcagttcgcctccgcctggcgtcgaacccgtgagcatgacaactggcgcacagggtccttaccaagtgacctatctcgtaccaagttgttgttgatgggagtagtgtatattatccattcatcctcgctctgtttttgtttctttttgtttcaatttatctattttcagtttgccaaatcctcgtgtttactgtcttgtttgtgtaaaaaaacagctgccatgtatcaagcgtaaagatgaatgacaagaaaaactattccgagaagacactcatgaagaaaacgtagtgttttgagctcctctcgctcttgttttgacagcttcaattttgtgaagtatgcccttttatgatttctcgatctttttttttgtgcaccagtgatttctcgatctagaaacaatatttagagtcaaggccagtgaggaaaatagtattcaggtttttcttttgtggctctttcttctccggcaaatcacaatagctttagtgacaaacttcaatatttaactaaaggatacgtattgttgcaacttagattctgatttattttccttccgtttagccagtgtaataaaattcaatcccttaagagcacctttaatggttagtatctacagataagttcctaaactaaaaagtaataataatctaaacaaataaaattgtataattaaaagttaatgcttttaaaaagtctagaccatttacacatgaccatctgtctagtgtgtatgagaaccttagctttaaatggaagtgctcttagagcatgattaacccggagttcttagggaaaggttaatcatggtctatacatgttacaaaaagggttctcaccgttagggttgtcctaatgaaagatgttcaatttgggttacccgaaccgaaactttaccggaaatccggtttggtgcatctgaacgtctgtaaaaaaaaagaagagtattccgagtctttgacattcaatcaaaattccacgtgtgacactcgttaagtcggccaattttttgtaccgtaaatgggcttttctgtcaaggtccatataagtccactttttatgatgtcattgtcatgagcccatccccaatcgtgataaatctcgtaaaccaatcaatacacgccacgtcattgacaagtagaactcgcgccacgtgagccgcgggtcaagcggtcatcatcgtccgacttggtatattccctcgtgttatcttccagaacaatatagagagagaagagaaccaattaaaaagactcatccaaacgaatctcagccgtccattcctcaccctattgttttctgttttcctctcttcttcttctcgctcgacaaatcgtaactaacaccttcacgaaaccgtcgtgagaaaccctaaaatcATGGCGAAAGAGATTCAATCTCAATCTCCTCCGTCGCCTACGACCACCGGAATCACGTTTTCTTCTTCTTCCTCTTCGCCGTCTCTGTCAATGATGCTATCCTCCACCAACGCGTTCTCGTTACTCTCGAAAGGGTGGAGGGAGGTGCGGGACTCAGCAGACGCGGATTTGCAGCTGATGCGGAAACGAGCGAACTCCGTTAAGAATCTAGCGTCGACGTTCGACCGCAAGATCGAGAATTTCCTCAACAACTCGGCGATGTCTGCGTTTCCCGTCGTGTCGTCGTCTCCGTCGGCGTTTGGCAATGAGATTGGGATCATGAAGAAGCTTGAGCCGAAGATATCGGAGTTCCGCAGGGTTTATTCGGCTCCGGAGATCAGCCGTAAGGTGATGGAGAGGTGGGGGCCAGCGAAGGCGAAGCTGGGGATCGATTTGTCGGCGATTAAGAAGGCGATTGTGTCTGAGATGGATCTGGATGAGCGGGAAGGGGTTTTGGAGATGAGGAGACGGCGGGATAGGGATAGGTTTAGGGAGTTTTACGCGGAGGGAGAGGGAGAAGGTAGTTTTGGAGATTGGAAGCCGATTAGGTCTTTGAAGAGTAGGTTTAAAGAGTTTGAGAAACGAAGCTCCTTAGAGTTATTGATTGGATTCAAGAACAGTGAGCTTGTGGAGAAGCTCAAAGCCAGCTTTCAATCACTTTATAAAGAAACTGATGAGGCCAAGGATGTTCCACCACTGGATGTACCTGAACTTTTGGCATCTTTGGTTAGGCAGTCAGAACCGTTCCTTGATCAGATTGGTGTAAGAAAGGATTTGTGCGACCGAGTAGTAGAAAACCTTTATAAATGCAAGAGCCAACACCTCTGGCGCCTGCCCTCTGCACAAACATCTGATTTAGTTGAAAATGATAACCATGTTGATGATTTGGATACGAGGATAGCCAGTGTTCTTCAGAGCACAGGACACCATTACGATGGTGGCTTTTGGACTGATTTTCTGAAGCCTGAGACGCCAGAAAGCAAAAGGCACGTGGCAATAGTTACAACAGCTAGTCTCCCGTGGATGACCGGAACAGCTGTAAATCCCCTGTTCCGAGCTGCCTATTTAGCAAAATCTGCAAAACAGAGTGTCACACTCGTGGTTCCTTGGCTCTGCGAATCTGATCAAGAACTAGTTTATCCAAACAATCTCACCTTCAGTTCACCTGAAGAACAAGAGAGTTATATACGTACATGGTTGGAGGAAAGGATTGGTTTCAAGGCTGATTTTAAAATCTCGTTTTACCCCGGAAAGTTTTCAAAAGAAAGGCGCAGCATATTTCCTGCTGGTGACACTTCTCAATTCATACCGTCAAAAGATGCAGACATTGCTATTCTTGAAGAGCCTGAACATCTCAACTGGTATCACCACGGCAAGCGTTGGACTGATAAATTCAACCATGTTGTTGGAATTGTCCACACAAACTACTTAGAGTACATCAAGAGGGAGAAGCATGGTGCTATCCAAGCATTTTTTGTGAACCATGTAAACAATTGGGTCACACGAGCATATTGCGACAAGGTTCTTCGCCTCTCTGGAGCAACACAAGATCTGCCAAAATCTGTTATATGCAATGTCCATGGTGTCAATCCCAAGTTCCTTATGATTGGGGAAAAAATTGCTGAGGAGAGTTCCCGTGGGGAACAAGCTTTCTCAAAAGGCGCATACTTCTTAGGAAAAATGGTGTGGGCTAAAGGCTACAGAGAACTAATAGATCTGATGGCTAAACACAAAAGCGACCTCGGGAGCTTCAATTTAGATGTGTACGGAAACGGTGAAGATGCAGTCGAAGTCCAACGTGCAGCACAGAAACTCGATTTGAATCTCAATTTCCTCAAAGGAAGGGACCATGCGGACGATGCTCTTCACAAGTACAAAGTGTTCATAAACCCGAGCATCAGCGATGTTCTATGCACAGCAACCGCGGAAGCACTAGCCATGGGGAAGTTCGTGGTGTGTGCAGACCATCCTTCAAACGAGTTCTTCAGATCATTCCCAAACTGTCTAACCTACAAAACATCCGAAGACTTTGTGAACAAAGTGAAAGAAGCAATGTCGAAAGAGCCGTTACCGCTCACCCCTGAGCAGATGTACAATCTTTCTTGGGAAGCAGCGACACAGAGATTCATGGAGTATTCGGATCTCGATAAGATCTTAAACGATGAAGACGGAGGAAAAAGGATGAGAAAATCAAGATCGGTTCCGAGCTTTGACGAGGTGATCGATGGAGGATTGGCGTTCACACACTATGTTCTCACAGGGAATGATTTCTTGAGACTATGCAGTGGAGCAACACCAAAAACAAAAGACTATGATAAGCAACATTGCAAGGATCTGAAACTTGTTCCACCTCAAGCTCACAAGCCAGTCTATGGCTGGTAGatgttttgccaacagtgattgctttttagatttgcttattagtaaaccactactacgtttgttatttgctatgatagttgtttaaggtcgtttgttaatagataggtactagggaagatcattttgtgggctattttgtagagtattaaatacaaagcgtatttaatgccatatgtaaaaatttactctccctgtttcaatatataaatagctttgaaagcttttttttgtttcataatataaattgttttgaaagtccaatgcaaaagagtaaaatgataaaataatatttaaacaaaa

**>BnaA06g04900D_BnaA06.FATB (up_Stream_Len 1500 Down_Stream_Len 300)**

attctaaagttgctcacgtatatcaagaaaaataataatatttacttttatctattagtattgtttggatttattttttttggcatcatctagtttttttttctaattagtttaaagttttgtttgcttcccaataactttcaaccaatttatatatttacatttgatttttttgtgttaatttaaagtaaaatgataaaatgtattagttaaagttaaaagatcaattttgtattaaaaaaaactaaaacatcagtttttgtatacataaagaatactatgaattaagtggtgaataatcaaagagagctctgtagtttattaaaggtttgagtaactttgaaaatcactaaaattacacaacgaatggcaaaaacaaggagtaacaaaataagcataaaaatacaaatgcatcactcagtgaatgcagcttcggttagttggatctacaaaccgaatggaaatggtgaacataggccacaacaaattaaaattacagctagctagctaagatattgatagacaaggtgatactagaccccctttttttttggtaaaacggtagagttattattcatacgcatacatcataaatttccaacataaatcgtaaattaccaacattttcatatacataatcagaagcaaactatgtggacacttgacaaagtatgcaatgtatattcaatatcacattctccaaaatgcaacgcatcactctaaacgctcagctctatccaaagagtcgcatgcttacacattcgcaaccgtggaggatactccaaaagactacgatcccaaaggacaacctatacaattgtggagagtgacaaagaagggagagcatatgaatggataatactagcactgcatagcttaacttgtatcgtttttttctccttaggttagtacttagtaggtatgttttacaaaaattaatttctatgaattttaaatataatataaaataatatgttttaggtgaaacaaatttataagtccaacggtggacttcacgttctacaaaaaaaacgtatagttaaacgaaccaacaaaataaactgttagaaatgcataatgttaggttttgtataaatgttatgtttcaatttgagctttgataaaatacacacgagtaaagaaagagataagatgcacatgtaccttgtttgttgtgcactcagcccactcagctattattactaaaacgtcggtgccaaagttgacaattctctgctaaatacaaaatctgatatacgtctctttctccacaacaatatgttgattggttagtgtaattagcaatcctcacatatagggaggaaatcaaatattcaaatccaaatgaaatttccacggaagcaagtaatcaagtcttgcgtgcttacataacgagtgaccaataatataaaaaagaattgaattagattagccttagttaggttaacaatcttttaacaagaaaagggtataattggaaatacaagaaaatttaaaaatATGGTTTTGAAACTACGAGAAGGAAGGAGAAAGGAAGAAGAAGAAGATGAAGGGGAATGCAATTTATATAAGAAAAGGCCTCTCTTTAAACAAAAGGAGGAGAGGAGAGGAGATCGTGAGGGAGGCACAACCGAAGAAGTAGGGACTTTGGAGAAAATTAGCGTTACCATTTTTGAGATTTTCATCCTCCATTCTACACCTGAAGTGTCTTTGAAACGCTCATCTCCTCAAGCCATGGTGGCCACCTCAGCTACATCCTCATTCTTCCCTCTCCCATCTTTCCCCCTCGACCCCACCGCAAAAACCAACAAAGTCACCACCTCCACCAACTTCTCCGGCCTCTCCCCCACTCCAAACTCCTCCGGCAGGATGAAGGTTAAACCAAACGCTCAGGCCCCACCCAAGATCAACGGCAAGAGAGTCGGTCTCCCTTCTGGCTCGGTGAAGCCTGATAACGAGACGTCCTCACAGCATCCCGCAGCACCGAGGACGTTCATCAACCAGCTGCCTGACTGGAGCATGCTTCTTGCTGCAATAACAACCGTCTTCTTGGCGGCTGAGAAGCAGTGGATGATGCTTGACTGGAAACCGAGGCGCTCTGACGTGATTATGGATCCGTTTGGGTTAGGGAGGATCGTTCAGGATGGGCTTGTGTTCCGTCAGAATTTCTCTATTCGGTCTTATGAGATAGGTGCTGATCGCTCTGCGTCTATAGAAACGGTTATGAATCATTTACAGGAAACGGCACTCAACCATGTTAAGACTGCTGGACTGCTTGGAGATGGGTTTGGTTCTACTCCTGAGATGGTTAAGAAGAACTTGATTTGGGTTGTTACTCGTATGCAGGTTGTCGTTGATAAATATCCTACTTGGGGAGATGTTGTGGAAGTAGATACATGGGTGAGCCAGTCTGGAAAGAACGGTATGCGTCGTGATTGGCTAGTTCGAGATGGCAATACTGGAGAAATTTTAACAAGAGCATCAAGTGTGTGGGTGATGATGAATAAACTGACAAGAAGATTATCAAAGATTCCTGAAGAGGTTCGAGGGGAGATAGAGCCTTACTTTGTTAATTCAGACCCAGTCCTTGCTGAGGACAGCAGAAAGTTAACTAAACTTGATGACAAGACTGCTGACTATGTTCGTTCTGGTCTCACTCCGCGTTGGAGTGACTTGGATGTTAACCAGCACGTTAACAATGTGAAGTACATCGGGTGGATACTGGAGAGTGCACCTGTGGGGATGATGGAGAGTCAGAAGCTGAAAAGCATGACTCTGGAGTATCGCAGGGAGTGCGGGAGGGACAGTGTGCTTCAGTCCCTCACCGCGGTTTCGGGCTGCGATGTTGGTAGTCTTGGGACAGCTGGTGAAGTGGAATGTCAGCACCTGCTCCGTCTCCAGGATGGAGCTGAAGTGGTGAGAGGAAGAACAGAGTGGAGTTCCAAAACATCAACAACAACTTGGGACATTACACCGTGAgagaaaaactcgcaaacatgggttctttggttcgtttgtaaactatactaccttgcttgcaaccaccactactcaaaaacagtttgggccacctttgtatatttttttggttcttatttttcttcttcttggaggtccctttttattatatttattttttcttttgggtgccagacaaaggcaaataactttcttatcctaatattatttaaatgtattttattttgggggttttgtagctgtttccacctaaacaaaagacgtgcatcatgtacttttgtgacctgtaaggaatgtaat

**>BnaC07g27360D_BnaC07.ATPXG2 (up_Stream_Len 1500 Down_Stream_Len 300)**

gggatcccaaacagtgcgactttggcctcactacatcgcaatggagcctggcttcttcctgccgcgagaacagagcaccaacttcaagtcctctccttcatcactacaatgagaagaggatgactatgagtggcatatcttggggaggctttccaacaaatatagtactggggatgtttatagtcacctgcgaggaaatggcgaagctgaagagtggaccaaagcaatctggacctcacggagcattccaaggcagagctttcatggatggttggtggcgctcaataggctcccgacgcgagataggttaataagttggggcttacagattccttctctgtaataattgtgatgaatccagggatcatctttattgggactgcccattctccttcgcactatggactctagtagctaatagatgccgaactactccgctgcgaagttgggtctcctcgcttcatcagatgtcgaccctacctcctccgtcaactgctcgatctctaactctccttggatggcaagctacactatactggatttggaacgagcgaaataaacggttgcatcaatctcaatttcgatcggtggattctctgttctccatcattgatcaccagttgaggaacaaaatcaataacttcagagagaacaaccctagacgctcctcagagatgttacaactatggtttcgttgatctatcacatgctccacatcgactctcctcccaccattgatcgtctctctgtttcttctacccgacaaatctctcggctcaccatctccatcttactaaagggctcaatcttttgggcttcctactgggcaaatatctaaggttaatgggttctaaaatatgagtttatattgggctgttgggctacttctactagatcattagtgcttttgtaaaacgtttttattctttctgcaattttaatatgaaggcagttacaaaaacaaaaaaaaaaaaatctagtgtgcttttctttttgtttatccagaaagcaagagaaatgagcatggaggtatcatccttttttaacgttttttcctgaccgctggattccactcgtgtaatcttaccatcgtctgagatttcgacacgtcattattttgttaaaagaaaaagcggtaattttgtcggccaccaacaacagtttatatgaaaccaataaaatacagcacaatgtggatcctatagcttgcgtggctcaacatgatcggtccacacgaaagaacttttgacaatgtagaagatgagcataacaaaataactcaattttgatttctggtttaaatgaagcaaaaatggcttagtcatagaaacacatggaaagattaccacgtgtcaacacagtaggtagttgttcaagacgaaagcaactcccgcgagttctttggaatcagttgcactctctcttctcagatctcttttcttatttataaaaaaaaaggcctctctgaaccacaaaacacaactagagagagaaagtacaATGGCGTCGGATGCAGGATCAGAGTCGATGGCGACGGTGGCTGCCAAAGCTCCGATAACAGCCGAGAGAAAGGTCCGAACTGATCTCGACAACCGTCTCCCTAAACCATATGTGCCACGAGCCATGGTTGCACCTGACATGGAAAACCTTGACGGGACAAGGGGACACAAGCACAACGACATGTCTGTTCTTCAACAGCACGTTGCTTTTTTTGATCAAGATGGTGATGGTATCATCTACCCATCCGAGACATTCCGAGGATTTAGAGCACTTGGTTTCAACGTGATCTTTTGTTTCTTTCTAACGTTCATCGTGCATCTTACCATGAGTTACGCCACATCGCCTACTTGGATGCCTACCTTTACGTTCCCGATTTACATCAAGAACATCCACAGAGCCAAACATGGAAGCGACACATCTACTTACGACACAGAAGGACGGTACATCCCGGCAAATCTGGAGAACATGTTTAGTAAGTACGCGCGTACAGTACCTGATAAGTTAACCCGTTACGAGTTGTGGCAAATGACTGAGGCTAACCGTAACGCATTCGATTTCTTTGGCTGGGCAGCGAGTAAAATGGAGTGGGGAGTACTCTACTTACTGGCTAAAGACGAGAATGGCTACTTATCCAAAGAAGCTGTTAGGAGATGCTTCGATGGGAGCTTGTTCGACTACTGCGCCAAGTCTAGAGCCGCTCCTAGAAAGGCTGATTGAtcaacatgctaacatgtttctcaattttctcggcagctaatgtctcttaaccgaaaacacgaatcagaccgtaacgttttgcttcgtaaatttcacatccttataatggttcttttggtttctatctttgacaagtgcgttgcttgacgtatatcgacgtgtcacggtgtttgtctatggatatcatagtatgaaagttttgtcgaacgttgtagaggatagtaatgcgaatagcatgcaacttggtgagtgaattaaacaagacagagggattatgttttgacatcaaatattgttatg

**>BnaC03g59130D_BnaC03.PDH_E1 BETA (up_Stream_Len 1500 Down_Stream_Len 300)**

ggtatatcgcggaaagttttgggctgcattttcattattatttaatagtctaagcttggagtgggcttggtgggtacccacatcagtcctggattcgaggaactaaattatcactacttggccagtctgggtttgggcttcggcccaagtggtttacatggtgggccataacagatgattggtccacccctggcattagtcggaaggtattccaaactcgggtcaggcagtgtggtacactttggggttagtccactctgtagcactaagtgcgttcctcccggagccgaccggatcagccgatagggtttataaaaaaaaaaaaaagcatataattgttgggtaatataaattcatacctattaaaaccaaccaaaaagtaaggttcggaataaataaataaggtctagccgacgtaaagttcgaatgaagaaaacgacatctagctatcttaaagaaatagagtcaagaaatgaaaaggttcaaccagcataaggtccaaaaagaaaaaaatgtccaactagcttagcataagccgtaaggtccgaaaagaaaaatattcaatcagtttaaggcctcaaattcaaaaacaaaatcgacttaaggtagtgctaggtttccatgctagcagaggatgcttctttcactgttacacacgtccatcctatgcggacactagacgggatcaccttaccccacggtaacagccagagccggacctaacgttacgggccctatacaaaattgaaattttatctaatttttagtaaaaaaaatattttatattttgtaaaagattatttttaaaataaataatcataaattttatctcataatctaactttacttaatatctttaacatttttatgttttatatagtatatatttatatgtatatagttttttttttaaattgggggccccatgaagtggaggccccattccaatgtttcatctcagtaggttcaagtccggctctggtaacagctcaacgcaacatcatggaagtataatgggaaaaacatgtgcaaaaagaacacctatccagaagaatttcccacttggtcaagcagtaagacattgacatatcattgggccggtacatttgaggcccatccccgcatccaaaaccaatgaaaggtcataagtattgttaaatgtaattgggttggtccggggtggttaagcgaacccgtcctaactcctctacaagcttgttcatggagttatatatactccacaatttagatacaaacacgtgtctactataatcgtaaatgaacacgtggcatacttatactggtttcatacaaaatagggggccacaggagattagaagtggtgcatggcgaagaaaacaatgtacaacaaacatgtggattggatcgaacaatagacacacaaatttttatttatttttacagaagaaatcaatttcacagctattctcactagtccttcgatttctccgcttcgtcactctccctggggaaaaaaaaATGGCTGCGAGAATCCATGGAGGAGCTGGAGCTGGAGCTGCTACGGCCTTGTCGACCTTTAATCCCAAGAAACTCGTTGCTCCTTCCCGCACCAATCTCCCAGCGAGGAGCAGCAAGAGATGCATTGTGGCTGCTGCTGGATCTGATGCGAGTAAGAGCCTCAGAGTTGGTCACTCTCAGAAATTGATTGCGAATGCTGCTGTTGCGACGAAGGCGGAGACATCTGCCACCACTGGCACTGGTCATGAACTACTGCTTTTCGAGGCTCTTCAGGAAGGTCTGGAAGAAGAGATGGACAGAGATCCACATGTATGTGTTATGGGTGAAGACGTTGGCCATTACGGTGGCTCCTACAAAGTAACCAAAGGCCTTGCTGATAAATTTGGCGACCTCAGGGTTCTCGACACTCCTATTTGTGAAAATGCATTCACCGGTATGGGAATTGGAGCTGCCATGACTGGTCTAAGACCTGTCATCGAAGGTATGAACATGGGTTTCCTCCTCCTGGCCTTCAACCAAATCTCCAACAACTGTGGAATGCTTCACTACACATCTGGTGGCCAGTTCACAATCCCGGTTGTCATCCGTGGTCCCGGTGGAGTGGGACGCCAGCTCGGCGCTGAGCATTCGCAGCGGCTAGAATCTTACTTCCAGTCCATTCCTGGGATCCAGATGGTTGCTTGCTCGACTCCTTACAACGCCAAAGGGCTGATGAAAGCCGCGATCAGAAGCGAGAACCCTGTGATTCTGTTTGAGCACGTTCTGCTTTACAATCTCAAGGAGAAGATACCGGACGAAGAGTACATTTGTAATCTTGAAGAAGCTGAGATGGTCAGACCTGGAGAGCACATCACCATCCTCACTTACTCGCGGATGAGGTACCATGTGATGCAGGCAGCTAAAACTCTGGTGAACAAAGGGTATGACCCTGAGGTTATCGACATCAGGTCGCTGAAACCGTTTGATCTTTACACGATTGGGAACTCGGTTAAGAAAACGCACAGGGACGTTCCTACACCGTACGCTGGTACACTGGAGGAGTGGACCGTTGTTCAACCGGCTCAGATCGTGACCGCCGTTGAGCAGCTTTGCCAGTGAaattcatagttttatcggtgagccattatttaccatttacctctttatttaccttctcttgagcttaattagttcgtaagaatttgtctaagttgtttgggttttgttaacgtttgttgccccttttgttgtgtgtttggaatatggctttgaaactcataatgtttcctttgttaatctatctcacacatttttatttttatcctttgaattttcattaccttttgctatgaacacacacacaacgcattctgttataaatgttattttcttgacacttacgttaataaacacatacaa

**>BnaA06g04170D_BnaA06.DSI-1VOC (up_Stream_Len 1500 Down_Stream_Len 300)**

gtttgacattgcaacgggttctaactctagactgataatggataatcttacctacaggtgattggtatgtatgtgggagtagcaacggcgggagtattcatcatatggtacacacacagcagcttcatggggatagacttgagccaagacggtcacagccttgtgagatattcacagctatctcattgggggcaatgctcttcatgggaaggcttcaaagtctctcctttcacagctggctctcagacattctcgttggacacaaacccgtgcgagtatttccatcaggggaagatcaaagcatccacactctccctctcggtgttggtagccatagaaatgttcaactccatgaacgcactctcagaagacactgaaacaccctagcattactattagcttacaattcactacgtttcttaactcagcctcgttgcttattttaatttttctaaacggtcattttttggttagataaaaaaacgttataacttgttccaaaactaaacccgaaatagagatgaatgttgtttcaaccaaccttcaatgttttgtttctttactttatttttctttattcaacgttttgtttttttactttgtataatcttttataatataaaataaatttctttgaagatggaggattatcacagcacaacttttgattaagattaatttaacataatgttggctaacaacacagcagaaaatgatacaacagtcgtggatttactcatctccgtcgatttaacctccgaaaccgtagagagtgcgaccttgcctcttcaaagcataaaccacatccatagcagtgacagtcttcctcctggcgtgctcggtgtaggtaacagcgtcgcggatgacattctcgaggaagatcttgaggactcctctggtctcttcgtagatcaaaccgctgatacgcttgacaccacctcgacgagcaagacgacgaatagcaggcttggtgataccttggatgttatctctaagcaccttcctgtgcctcttggctcctccctttcccaatccttttcctcctttccctcttcctgacatcttctaacaagtatttaaggaaaacaatagagtaaatttctgatggtgaaaaagagaagagattgacgtatatgtataaagagggagaagattgtaagccgttcgattcaatcttctttcaatggttagagattgcgatccgcgtgaaagctttgtaacaaatataatacgtcgattataacacatcgacggtggtagattgcttcctttagtttaagttgacaaaacgaaatcgccacgtcacctccgccgaagttaaggcttttaaaagcccgtttgtaaatcataggcccaactataaatggacgaatgctcacgtggcagtccgcaaaggagcatagacacgtaatcgtccacgtgttgagctcgccgtaaagagagatcttgttagtgtatttgtaatagaccgagttggagaatcacaaggattgaaaataaATGGCGTCGAATGTCATAAAACCAGCGTATGCTTACACGGTTGTGTACGTTAGGGACGTCGCGAAATCCGTAGAATTCTACTCGAGAGCCTTTGGTTACAACGTTCGTCGTCTTGACGAGTCCCACAGGTGGGGGGAGCTAGAGAGCGGGCAGACAACGATAGCATTCACACCGCTTCACCAGCATGAGACGGATGACCTAACCGGTAAAGTTCAGTCGTCTACGCGTTCAGAACGTGAGAGAGCACCCCTCGAAGTCTGCTTCTGTTATGCTGATGTTGACGCGGCTTTCAAGAGGGCTGTGGAGAATGGTGCGGTGGCTGTGAGCGAGCCGGAGGACAAGGAATGGGGCCAAAAGGTTGGATACGTTCGAGACATTGATGGCATCGTTGTACGCATCGGAAGCCACGTTAAGTCATATAAACCTTGGACAAGTTTCGGGTTTTCTCTAGCTATCAATCTTAATCTTCCAGTTGTTCCACAGCTCAACAATATAACAAGCAAAAAAAGGCAATAAagtactttgaaactttttctttttgtccttttgcagtctttatatgtttgtgaatgcctctctttgcaaaaaagtgtgattgcctgattggccacctgtcatagtgaaagctaagccaataatgcaatgcgtatttggtctttgaaaccaaaatcaagatgagacgcagttatccgctacaaataatattgtaatcacccgggaccaggagaaagggatcctatatataaaactggaatcccactttgctaaacattttagaaattgttagatgtatatattaaacatacaaaaattact
